# Lysosomal protein surface expression discriminates fat- from bone-forming human mesenchymal precursor cells

**DOI:** 10.7554/eLife.58990

**Published:** 2020-10-12

**Authors:** Jiajia Xu, Yiyun Wang, Ching-Yun Hsu, Stefano Negri, Robert J Tower, Yongxing Gao, Ye Tian, Takashi Sono, Carolyn A Meyers, Winters R Hardy, Leslie Chang, Shuaishuai Hu, Nusrat Kahn, Kristen Broderick, Bruno Péault, Aaron W James

**Affiliations:** 1 Departments of Pathology, Johns Hopkins UniversityBaltimoreUnited States; 2 Departments of Orthopaedics, Johns Hopkins UniversityBaltimoreUnited States; 3 Department of Oral and Maxillofacial Surgery, School of Stomatology, China Medical UniversityShenyangChina; 4 UCLA and Orthopaedic Hospital Department of Orthopaedic Surgery and the Orthopaedic Hospital Research CenterLos AngelesUnited States; 5 Departments of Plastic Surgery, Johns Hopkins UniversityBaltimoreUnited States; 6 Center For Cardiovascular Science and Center for Regenerative Medicine, University of EdinburghEdinburghUnited Kingdom; Maine Medical Center Research InstituteUnited States; Loma Linda UniversityUnited States

**Keywords:** perivascular stem cell, mesenchymal stem cell, osteogenesis, adipogenesis, CD107a/LAMP1, exocytosis, Human, Mouse, Rat

## Abstract

Tissue resident mesenchymal stem/stromal cells (MSCs) occupy perivascular spaces. Profiling human adipose perivascular mesenchyme with antibody arrays identified 16 novel surface antigens, including endolysosomal protein CD107a. Surface CD107a expression segregates MSCs into functionally distinct subsets. In culture, CD107a^low^ cells demonstrate high colony formation, osteoprogenitor cell frequency, and osteogenic potential. Conversely, CD107a^high^ cells include almost exclusively adipocyte progenitor cells. Accordingly, human CD107a^low^ cells drove dramatic bone formation after intramuscular transplantation in mice, and induced spine fusion in rats, whereas CD107a^high^ cells did not. CD107a protein trafficking to the cell surface is associated with exocytosis during early adipogenic differentiation. RNA sequencing also suggested that CD107a^low^ cells are precursors of CD107a^high^ cells. These results document the molecular and functional diversity of perivascular regenerative cells, and show that relocation to cell surface of a lysosomal protein marks the transition from osteo- to adipogenic potential in native human MSCs, a population of substantial therapeutic interest.

## Introduction

Within mammalian white adipose tissue (WAT), a perivascular population of mesenchymal progenitor cells is well documented with respect to multipotency and tissue renewal capabilities (Corselli et al., 2012; Kramann et al., 2016). The ability of human WAT-resident perivascular cells to differentiate into bone-forming osteoblasts and incite or participate in bone repair is also well known (Askarinam et al., 2013; Chung et al., 2014; James et al., 2012a; James et al., 2012b; James et al., 2012c; Lee et al., 2015; Meyers et al., 2018b; Tawonsawatruk et al., 2016) (see (James et al., 2017; James and Péault, 2019) for reviews). The bulk of WAT-resident perivascular cells with mesenchymal progenitor cell attributes reside in the *tunica adventitia* – the outer collagen-rich sheath of blood vessels (Corselli et al., 2012; James et al., 2012a; West et al., 2016). Microvascular pericytes, although less frequent in absolute numbers, also demonstrate progenitor cell attributes (Chen et al., 2013; Crisan et al., 2009; Crisan et al., 2008). With several recent studies from our group in human (Ding et al., 2019; Hardy et al., 2017) and mouse WAT (Wang et al., 2020), it is clear that perivascular cells, including those found within the *tunica adventitia* (adventitial cells or adventicytes), demonstrate more phenotypic and functional diversity than previously understood.

CD107a (lysosome-associated membrane protein-1, LAMP1) is a member of a family of structurally related type one membrane proteins predominantly expressed in lysosomes and other intracellular vesicles (Carlsson et al., 1988; de Saint-Vis et al., 1998; Defays et al., 2011; Ramprasad et al., 1996). CD107a is far less frequently expressed on the cell surface, which is the result of both trafficking of nascent protein to the plasma membrane as well as the fusion of late endosomes and lysosomes to the cell membrane (Akasaki et al., 1993; Dell'Angelica et al., 2000). In inflammatory cells, surface CD107a reflects the state of activation (Janvier and Bonifacino, 2005) and has been implicated in cell adhesion (Kannan et al., 1996; Min et al., 2013). In separate reports, CD107a has been described in intracellular vesicles in both osteoblasts and adipocytes (Bandeira et al., 2018; Solberg et al., 2015), yet beyond this, essentially nothing is known regarding CD107a in mesenchymal stem cell fate or differentiation decisions.

Here, antibody array screening of FACS-defined stromal vascular fraction (SVF) perivascular cells identified several novel cell surface antigens, including CD107a, enriched within subpopulations of human adventicytes and pericytes. Flow cytometry and immunohistochemical analyses confirmed that cells with membranous surface CD107a expression reside in a perivascular microanatomical niche within WAT. CD107a^high^ cells represent an adipocyte precursor cell, while CD107a^low^ cells represent progenitors with increased osteoblast potential. CD107a trafficking to the cell surface was observed to occur during early adipocyte differentiation – results confirmed by single-cell RNA sequencing datasets from mouse and human adipose tissues. Upon transplantation into immunocompromised rodents, CD107a^low^ cells robustly induce bone formation, both in an intramuscular ectopic ossicle assay in mice and a lumbar spine fusion rat model. These results suggest that cell surface CD107a divides osteoblast from adipocyte perivascular precursors within human tissues.

## Results

### Identification of CD107a as a novel cell surface antigen expressed among adipose tissue (AT)-resident perivascular stem cells

To identify new markers that may define subsets of perivascular cells, a cell surface antigen screen (Lyoplate) was performed on previously defined perivascular cell fractions (Crisan et al., 2008; James et al., 2012c; Xu et al., 2019), including CD34^+^CD146^-^ adventitial cells and CD146^+^CD34^-^ pericytes after exclusion of non-viable, endothelial, and hematopoietic cells (PI^+^ CD31^+^ or CD45^+^ fractions) (Table 1). Several markers were confirmed to be highly expressed among both adventicytes and pericytes, including, for example, the progenitor cell and MSC marker CD90 (Thy-1) and the perivascular cell antigen CD140b (PDGFRβ). Novel markers to divide perivascular progenitors ranged broadly, including the endolysosomal protein CD107a (32% and 82% expression among adventicytes and pericytes, respectively). Another endolysosomal protein, CD107b, was also present on each perivascular cell fraction (13% and 46% expressing adventicytes and pericytes, respectively). Other markers noted to be expressed differentially in subsets of adventicytes and pericytes included CD98, CD140a (PDGFRα), CD142, CD165, CD200, and CD271 (NGFR) (Table 1).

**Table 1.** Surface antigens expressed within human adventitial cells versus pericytes. Results derived from Lyoplate analysis of CD34^+^CD146^-^CD45^-^CD31^-^ adventitial cells or CD146^+^CD34^-^CD45^-^CD31^-^ pericytes.

CD marker	Protein name	Frequency in adventitial cells (CD34^+^CD146^-^CD45^-^CD31^-^)	Frequency in pericytes (CD146^+^CD34^-^CD45^-^CD31^-^)
CD90	Thy-1	97%	70%
CD91	Low-density lipoprotein-related receptor	97%	61%
CD95	Fas receptor (TNFRSF6)	42%	22%
CD98	Large neutral amino acid transporter (LAT1)	17%	65%
CD105	Endoglin	47%	14%
CD107a	Lysosomal-associated membrane protein 1 (LAMP1)	32%	82%
CD107b	Lysosomal-associated membrane protein 2 (LAMP2)	13%	46%
CD130	Interleukin six beta transmembrane protein	39%	61%
CD140a	Platelet-derived growth factor receptor alpha (PDGFRA)	82%	13%
CD140b	Platelet-derived growth factor receptor beta (PDGFRB)	97%	34%
CD142	Tissue factor, PTF, Factor III, or thromboplastin	47%	75%
CD147	Basigin (BSG)	99%	99%
CD151	Raph blood group	71%	100%
CD164	Sialomucin core protein 24 or endolyn	91%	97%
CD165	AD2	77%	21%
CD271	Nerve growth factor receptor (NGFR)	64%	10%

Next, previously derived transcriptomics data on human perivascular cells were analyzed to confirm *LAMP1* gene expression, encoding CD107a. Using WAT-derived pericytes (n = 3 samples, GEO dataset: GSE125545) or adventitial perivascular stem cells (n = 3 samples, GEO dataset: GSE130086) (Xu et al., 2019), high expression of the *LAMP1* gene was confirmed (mean FPKM values of 9.576 and 9.619, respectively).

**Figure 1.**
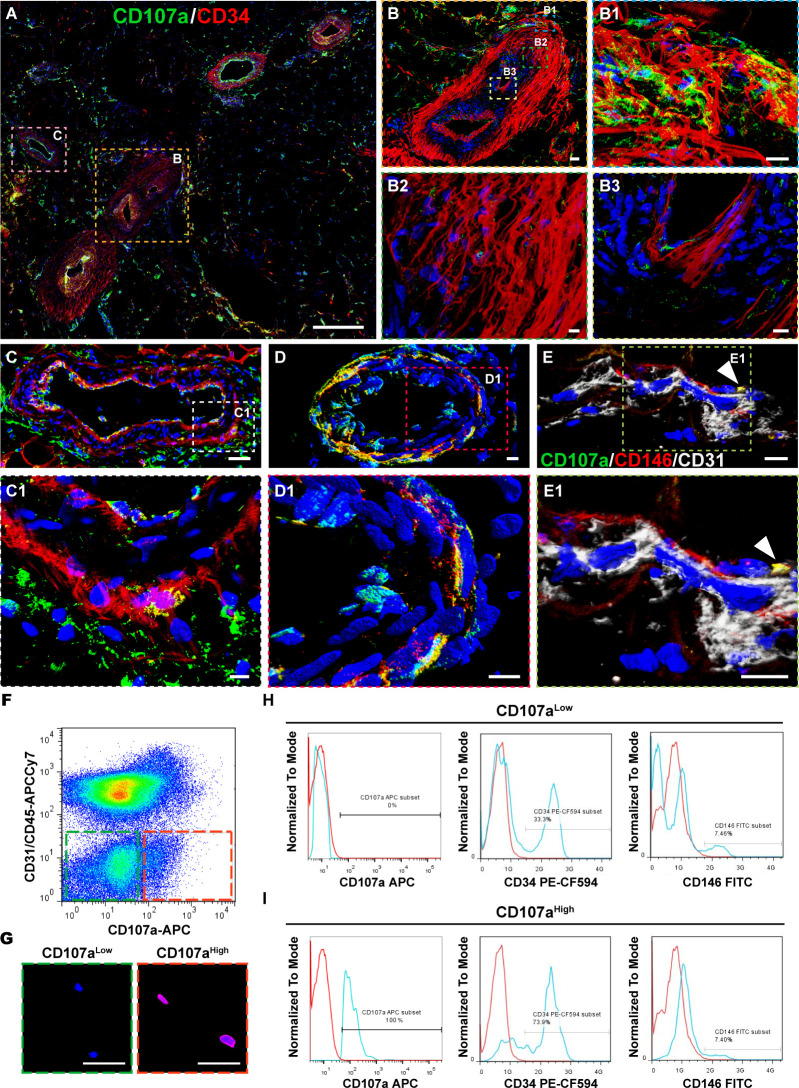
Perivascular CD107a expression typifies a subset of perivascular cells within human subcutaneous white adipose tissue (WAT). Immunofluorescent staining of CD107a (green) and CD34 (red) in human adipose tissue. (**A**) Tile scan. (**B**) Larger artery in cross-section, including (**B1**) outer tunica adventitia, (**B2**) inner tunica adventitia, and (**B3**) tunica media and intima. (**C**) Larger vein in cross-section, including (**C1**) high magnification of vessel wall. (**D**) Smaller caliber artery in cross-section, including (**D1**) high magnification of vessel wall. (**E**) Capillary in longitudinal cross-section, and (**E1**) high magnification. (**F**) Representative FlowJo plot to demonstrate partitioning CD107a^low^CD31^-^CD45^-^ and CD107a^high^CD31^-^CD45^-^ fractions from human stromal vascular fraction (SVF). Frequency of CD107a^low/high^ cells across samples is shown in Supplementary file 1 (N = 8 samples). (**G**) Confirmatory immunofluorescent staining of FAC-sorted CD107a^low^ and CD107a^high^ mesenchymal cells (CD31^-^CD45^-^PI^-^ cells). (**H,I**) Representative flow cytometry analysis of freshly isolated CD107a^low^ and CD107a^high^ mesenchymal cells, including CD107a, CD34, and CD146. Frequency of expression is shown in relation to isotype control (blue vs. red lines). Frequency of CD34^+^ and CD146^+^ cells across all samples is shown in Supplementary file 2, 3 (N = 4 samples). Scale bars: 500 μm (**A**), 50 μm (**B,C,G**) and 10 μm (**B1–B3,C1,D,D1,E,E1**).

Spatial localization of CD107a was next assessed by immunostaining of subcutaneous WAT (Figure 1A–E, n = 3 samples). CD107a immunoreactivity was found most frequently within the outermost layers of larger arteries (Figure 1B) and veins (Figure 1C). Within arteries, the outer *tunica adventitia* showed a high frequency of CD107a^+^CD34^+^ cells (Figure 1B1), while the inner *adventitia* showed predominantly CD107a^-^CD34^+^ cells (Figure 1B2). The smooth muscle media largely did not show CD107a immunoreactivity (Figure 1B3), which was confirmed by dual immunohistochemistry for CD107a and αSMA (Figure 1—figure supplement 1A). Co-expression with the recently described adventitial marker Gli1 was assessed (Kramann et al., 2016), which showed little overlap with CD107a immunoreactive adventitial cells (Figure 1—figure supplement 1B). Smaller caliber arteries (Figure 1D) and veins showed a high frequency of dual expressing CD107a^+^CD34^+^ cells within the *adventitia*. Capillaries within WAT showed some CD107a immunoreactive pericytes, which co-expressed CD146 but not CD31, and were present in an abluminal location (Figure 1E, appearing yellow). CD107a immunoreactivity within the perivascular mesenchymal niche was confirmed across other AT depots, including pericardial, perigonadal, perirenal, and omental human fat (Figure 1—figure supplement 2, n = 3 samples per depot). Small and medium caliber vessels showed perivascular immunoreactivity across all adipose depots.

**Figure 1—figure supplement 1.**
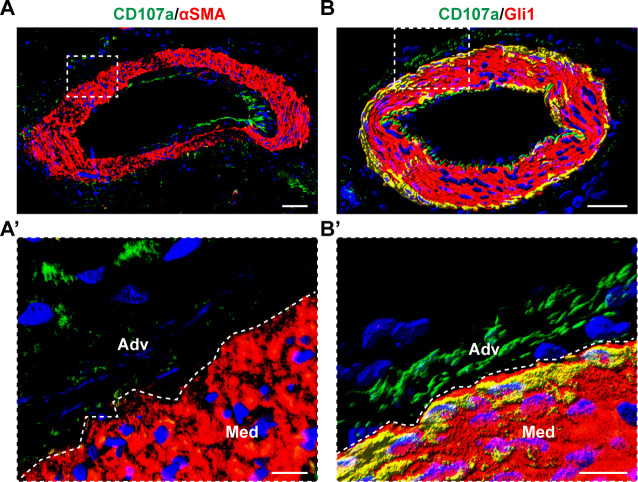
Comparison of CD107a expression with either αSMA or Gli1 in human adipose tissue. (**A**) Co-immunostaining of CD107a (green) and αSMA (red). (**A’**) High magnification image of outer vessel wall. (**B**) Co-immunostaining of CD107a (green) with Gli1 (red). (**B’**) High Magnification image of outer vessel wall. Overlap appears yellow. White scale bar: 50 μm (upper panels) and 15 μm (lower panels). Med: Tunica media; Adv: Tunica adventitia. N = 3 human samples examined.

**Figure 1—figure supplement 2.**
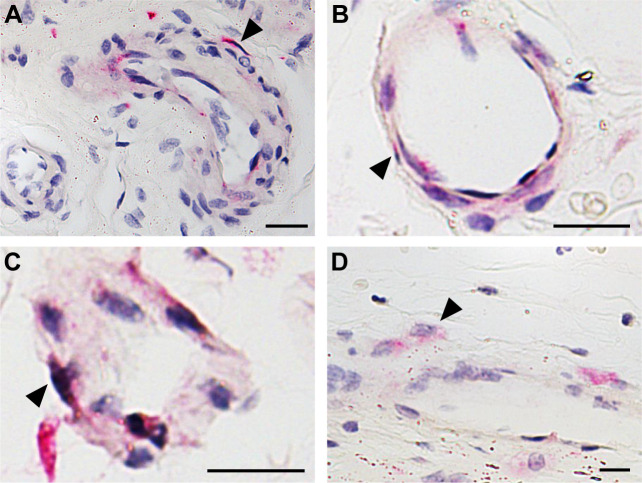
Perivascular CD107a immunohistochemical staining in diverse human adipose tissues. (**A**) Visceral pericardial fat, (**B**) visceral perigonadal fat, (**C**) visceral perirenal fat, and (**D**) omental fat. CD107a positive staining appears red (black arrowheads). N = 3 human samples per tissue depot examined. Black scale bar: 20 μm.

**Figure 1—figure supplement 3.**
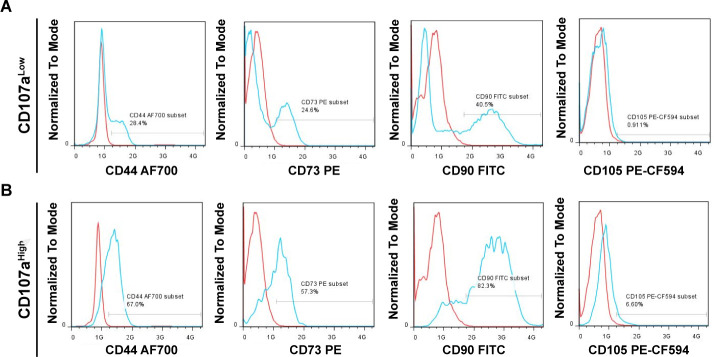
Flow cytometry of canonical human mesenchymal stem cell (MSC) markers among freshly isolated CD107a^low^**^/^**^high^ cells from a representative patient sample. (**A, B**) Expression of CD44, CD73, CD90, and CD105 within (**A**) CD107a^low^ and (**B**) CD107a^high^ cells. Frequency of expression is shown in relation to isotype control antibody (blue vs. red lines). Corresponding tabular data from N = 3 human samples is shown in Supplementary file 4.

Next, flow cytometry demonstrated a spectrum of CD107a membranous staining across the viable, non-endothelial/noninflammatory cells of human WAT (Figure 1F). The PI^-^CD31^-^CD45^-^ component of SVF was divided by FACS into CD107a^low^ and CD107a^high^ cell populations for further analysis (Figure 1F,G). Mean frequency of CD107a^low^ mesenchymal cells represented 33.75% of viable SVF, while mean frequency of CD107a^high^ mesenchymal cells represented 5.20% (Supplementary file 1). Flow cytometry analysis was next performed within CD107a^high^CD31^-^CD45^-^ and CD107a^low^CD31^-^CD45^-^ mesenchymal populations (Figure 1H,I). High expression of CD107a was confirmed by flow cytometry among freshly sorted CD107a^high^ cell preparations (Figure 1I). Concordant with histological observations, CD34^+^ and CD146^+^ cells were identified in both CD107a^low^ and CD107a^high^ cell fractions (Figure 1H,I, mean frequencies reported in Supplementary file 2, 3). These data confirmed that surface CD107a expression is present in both perivascular native MSC niches within WAT, and that surface CD107a could be used to prospectively purify mesenchymal cell subpopulations with disparate staining intensities.

Next, canonical markers of culture-expanded human MSCs were examined by flow cytometry in freshly sorted CD107a^low^ and CD107a^high^ cells, including CD44, CD73, CD90, and CD105 (Supplementary file 4, Figure 1—figure supplement 3). With the exception of CD105, all markers showed overall similar expression patterns across CD107a^low^ and CD107a^high^ cell populations (n = 3 samples per group). CD105 expression was disproportionately present within CD107a^high^ mesenchymal cells (mean frequency 0.44% and 8.12% among CD107a^low^ and CD107a^high^ cells, respectively).

### CD107a^low^ AT-derived stromal cells represent osteoblast precursor cells

**Figure 2.**
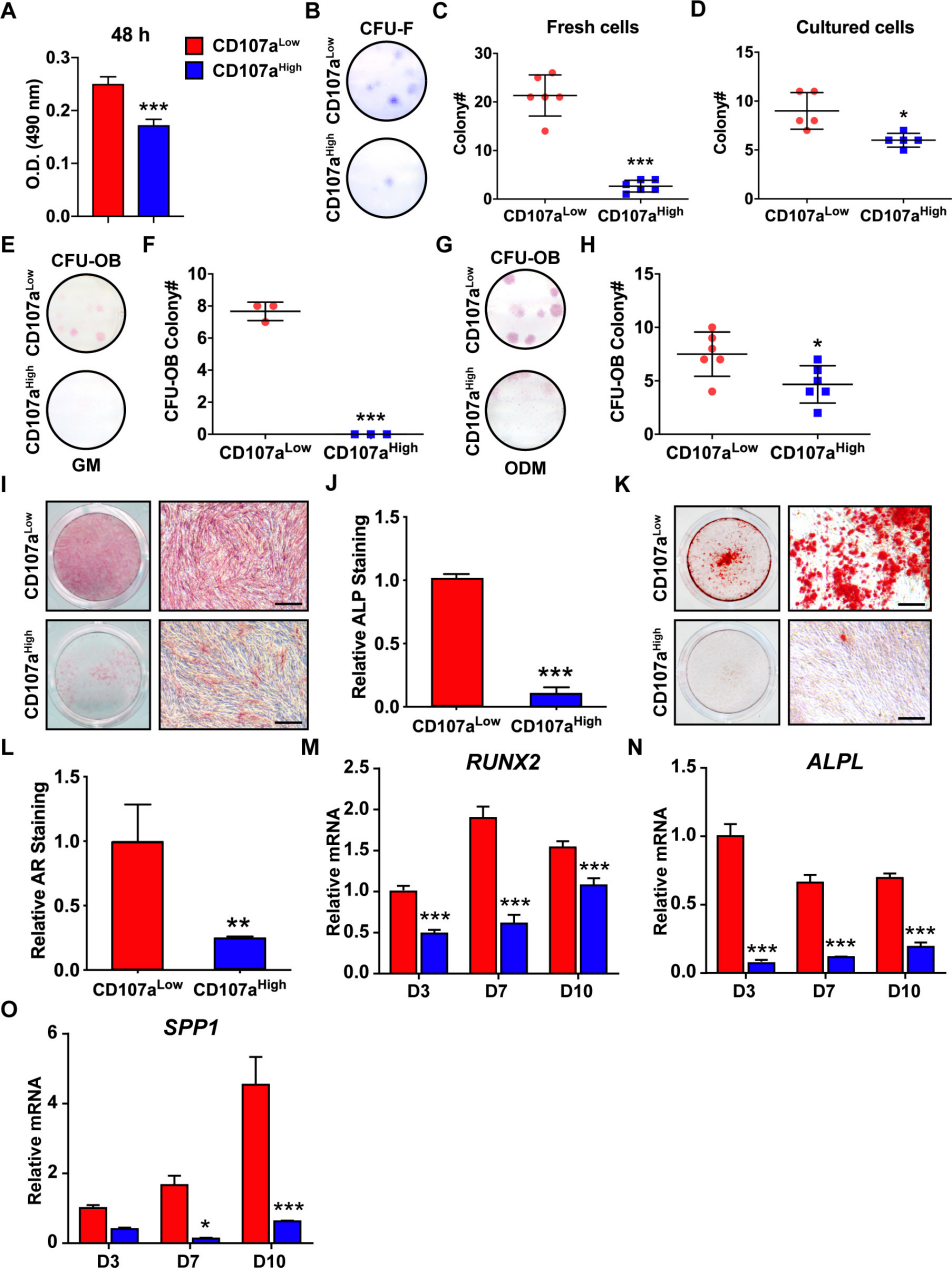
Stem/osteoprogenitor cell identity of CD107a^low^ mesenchymal cells. CD107a^low^CD31^-^CD45^-^ and CD107a^high^CD31^-^CD45^-^ cells derived from the same sample of human subcutaneous WAT were exposed to the indicated growth or osteogenic conditions. (**A**) Cell proliferation among CD107a^low^ and CD107a^high^ mesenchymal cells, by MTS assays at 48 hr. (**B–D**) Fibroblastic colony formation frequency (CFU-F) among human CD107a^low^ and CD107a^high^ mesenchymal cells, shown by (**B**) representative images among freshly isolated cells, (**C**) CFU-F quantification among freshly isolated cells, (**D**) CFU-F quantification among passage 4 cells. Whole well images are shown. (**E–H**) Osteoblastic colony formation frequency (CFU-OB) detected in human CD107a^low^ and CD107a^high^ cells. Experiments performed in growth medium (GM) (**E,F**) or osteogenic differentiation medium (ODM) (**G,H**). Whole well images are shown. (**I,J**) Alkaline phosphatase (ALP) staining and photometric quantification at d 10 of osteogenic differentiation among human CD107a^low^ and CD107a^high^ cells. Representative whole well and high magnification images are shown. (**K,L**) Alizarin red (AR) staining and photometric quantification at d 10 of osteogenic differentiation among human CD107a^low^ and CD107a^high^ cells. Representative whole well and high magnification images are shown. (**M–O**) Osteogenic gene expression among human CD107a^low^ and CD107a^high^ cells by qRT-PCR at d 3, 7, and 10 of differentiation, including (**M**) *Runt related transcription factor 2* (*RUNX2*), (**N**) *ALPL*, and (**O**) *Osteopontin* (*SPP1*). Osteogenic differentiation examined in N = 3 human cell preparations, and at least experimental triplicate. Dots in scatterplots represent values from individual wells, while mean and one SD are indicated by crosshairs and whiskers. In column graphs, mean values and one SD are shown. *p<0.05; **p<0.01; ***p<0.01 in relation to corresponding CD107a^low^ cell population. Statistical analysis was performed using a two-tailed Student t-test (**A–L**) or two-way ANOVA followed by Sidak’s multiple comparisons test (**M–O**). Experiments performed in at least biologic triplicate. Scale bars: 200 μm.

CD107a^low^ and CD107a^high^ cells were again derived from the CD31^-^CD45^-^ fraction of adipose tissue samples, and in vitro properties examined (Figure 2). Morphology of adherent CD107a^low^ and CD107a^high^ cells was broadly similar, with a fibroblastic shape (Figure 2—figure supplement 1). CD107a^low^ cells demonstrated a higher proliferative rate in comparison to CD107a^high^ cells (Figure 2A). Among freshly isolated cells, the vast majority of colony forming units-fibroblast (CFU-F) was identified within the CD107a^low^ cell fraction (Figure 2B,C). Among equivalent cells at passage 4, an enrichment in CFU-F was still observed in CD107a^low^ cells (Figure 2D). CFUs-osteoblast (CFU-OB) likewise showed a similar enrichment among CD107a^low^ cells. CFU-OB assays performed in growth medium showed alkaline phosphatase (ALP)^+^ colonies among CD107a^low^ cells only (Figure 2E,F). The same experiment performed in osteogenic differentiation medium showed an enrichment in CFU-OB among CD107a^low^ cells (Figure 2G,H). Among passaged cells in sub-confluent monolayer, osteogenic differentiation was next examined (Figure 2I–O). ALP staining and quantification demonstrated an enrichment among CD107a^low^ cells (Figure 2I,J). Bone nodule formation was likewise increased in CD107a^low^ cells as compared to CD107a^high^ counterparts (Figure 2K,L). Osteogenic gene expression across timepoints of differentiation likewise showed an enrichment for *RUNX2 (Runt related transcription factor 2)*, *ALPL*, and *osteopontin (SPP1)* (Figure 2M–O). Thus, the CD107a^low^ mesenchymal component of human WAT contains a precursor cell population with high osteoblastogenic potential.

**Figure 2—figure supplement 1.**
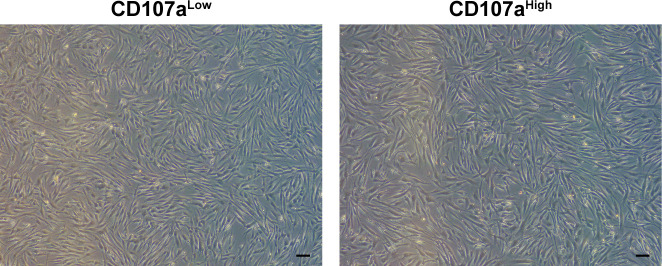
Representative morphology of confluent human CD107a^low^ and CD107a^high^ cells. Passage 4. N = 8 samples examined. Black scale bar: 100 μm.

### CD107a^high^ AT-derived stromal cells represent adipocyte precursor cells

**Figure 3.**
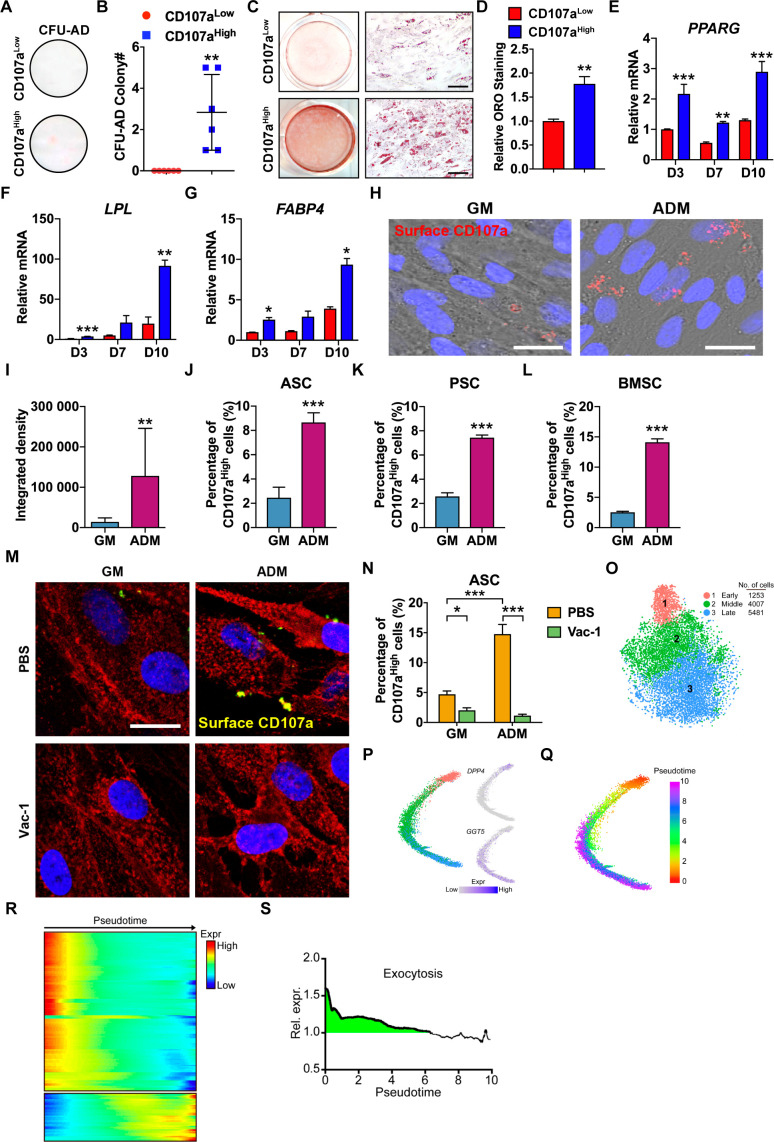
Adipoprogenitor cell identity of CD107a^high^ human mesenchymal cells and correlation to exocytosis during early adipogenic differentiation. (**A–G**) CD107a^low^CD31^-^CD45^-^ and CD107a^high^CD31^-^CD45^-^ cells derived from human subcutaneous WAT were exposed to adipogenic differentiation conditions. (**A,B**) Adipocyte colony formation frequency (CFU-AD) detected in human CD107a^low^ and CD107a^high^ cells. Whole well images shown. (**C,D**) Oil red O (ORO) staining and photometric quantification at d7 of adipogenic differentiation among human CD107a^low^ and CD107a^high^ cells. Representative whole well and high magnification images shown. (**E–G**) Adipogenic gene expression among human CD107a^low^ and CD107a^high^ cells by qRT-PCR at d 3, 7, and 10 of differentiation, including (**E**) *Peroxisome proliferator-activated receptor-γ* (*PPARG*), (**F**) *Lipoprotein lipase* (*LPL*), and (**G**) *Fatty acid-binding protein 4* (*FABP4*). (**H**) Immunocytochemical staining of membranous CD107a in the presence of growth medium (GM) or adipogenic differentiation medium (ADM) after 3 d using human, culture-defined adipose-derived stem cells (ASCs). CD107a immunoreactivity appears red, nuclear counterstain appears blue. (**I**) Photographic quantification of membranous CD107a immunofluorescence under GM or ADM conditions. (**J–L**) Induction of membranous CD107a expression after adipogenic differentiation across cell types, including (**J**) culture-defined human ASCs, (**K**) FACS-purified human perivascular stem cells (PSC), and (**L**) culture-defined human BMSCs, assessed by flow cytometry after 3 d under GM or ADM conditions. (**M,N**) Trafficking of CD107a to the cell surface during adipogenesis was inhibited after treatment with Vacuolin-1 (Vac-1, 1 μM), assessed by CD107a immunostaining (**M**) and flow cytometry (**N**) after 3 d under GM and ADM conditions. The cell membrane was labeled using Wheat Germ Agglutinin Conjugates (red), while overlap with CD107a immunostaining appears yellow, and DAPI nuclear counterstain appears blue. (**O**) Dimensional reduction and unsupervised clustering of human stromal vascular fraction (SVF) adipogenic lineage from subcutaneous WAT revealed three cell groups. (**P**) Trajectory analyses of human SVF adipogenic lineage, colored based on their unsupervised clustering identity. *DPP4* (early) and *GGT5* (late) expression were used to identify trajectory origin. (**Q**) Pseudotemporal cell ordering along differentiation trajectories. Pseudotime is depicted from red to purple. (**R**) Expression heatmap across pseudotime of genes associated with exocytosis. (**S**) Combined, normalized expression of exocytosis genes shows enrichment (>1) in early progenitors (green shaded area), while more differentiated cells show reduced average expression. Dots in scatterplots represent values from individual wells, while mean and one SD are indicated by crosshairs and whiskers. In column graphs, mean values and one SD are shown. *p<0.05; **p<0.01; ***p<0.001. Statistical analysis was performed using a two-tailed Student t-test (**B,D,I–L**) or two-way ANOVA followed by Sidak’s multiple comparisons test (**E–G,N**). Experiments performed in at least biologic triplicate. Black scale bar: 100 μm; white scale bar: 20 μm.

Converse experiments to assay adipogenesis were next performed among cell subsets with differential CD107a expression (Figure 3). Among freshly isolated cells, essentially all CFU-adipocyte (CFU-AD) were found within the CD107a^high^ cell population (Figure 3A,B). Next, sub-confluent CD107a^low^ and CD107a^high^ cells were propagated under adipogenic differentiation conditions (Figure 3C–G). Oil red O staining was significantly more abundant among the CD107a^high^ cells (Figure 3C,D). Adipogenic gene expression was next assessed along adipogenic differentiation (Figure 3E–G). All marker gene transcripts showed significant enrichment among CD107a^high^ cells in comparison to CD107a^low^ counterparts, including *peroxisome proliferator-activated receptor gamma (PPARG)*, *lipoprotein lipase (LPL)*, and *fatty acid-binding protein 4 (FABP4)*.

**Figure 3—figure supplement 1.**
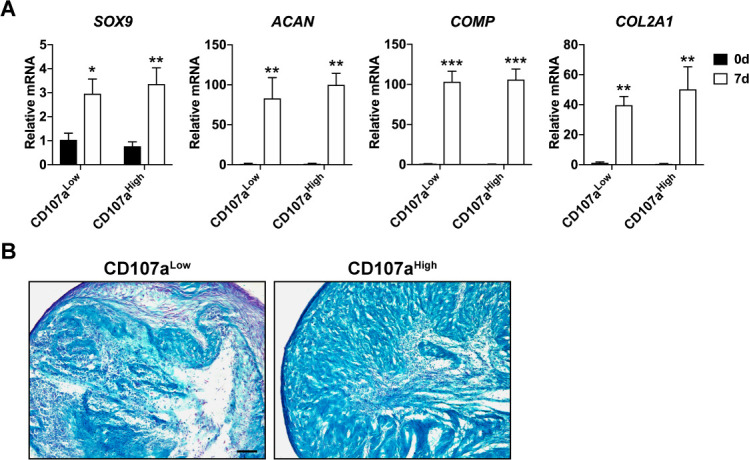
Chondrogenic differentiation among human CD107a^low^ and CD107a^high^ cells. (**A**) Chondrogenic gene expression among human CD107a^low^ and CD107a^high^ mesenchymal cells, including *SRY-Box transcription factor 9* (*SOX9*), *Aggrecan* (*ACAN*), *Cartilage oligomeric matrix protein* (*COMP*), and *Collagen type 2 A1* (*COL2A1*), assessed by qRT-PCR at 0 and 7 d of differentiation. (**B**) Chondrogenic differentiation, as assessed by alcian blue staining at 28 d of differentiation in high-density micromass culture. Black scale bar: 100 μm. *p<0.05; **p<0.01; ***p<0.001 in relation to corresponding 0 d sample. Statistical analysis was performed using a two-way ANOVA followed by Sidak’s multiple comparisons test. Data repeated in experimental triplicates.

**Figure 3—figure supplement 2.**
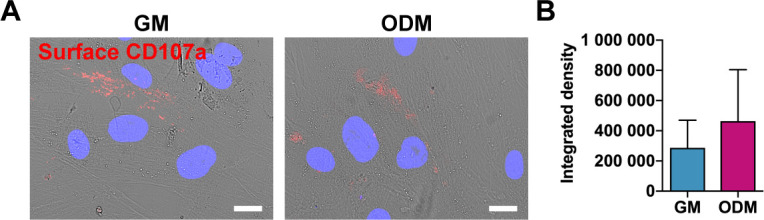
No change in membranous CD107a during osteogenic differentiation among human ASCs. (**A**) Immunocytochemical staining for membranous CD107a expression in the absence or presence of osteogenic differentiation medium for 3 d of differentiation. CD107a immunoreactivity appears red, DIC appears white. White scale bar: 20 μm. (**B**) Photometric quantification of membranous CD107a immunofluorescence with or without osteogenic differentiation. GM: Growth medium; ODM: Osteogenic differentiation medium. Data repeated in experimental triplicate. Statistical analysis was performed using a two-tailed Student t- test.

**Figure 3—figure supplement 3.**
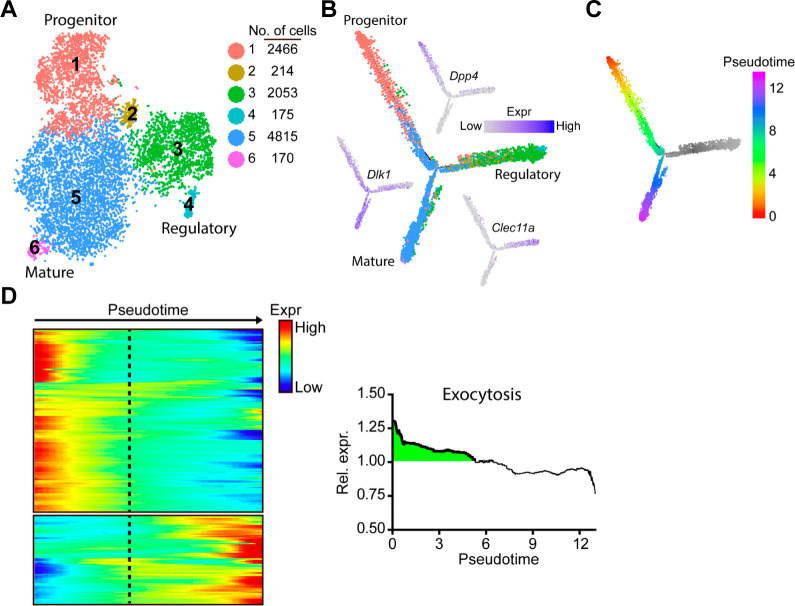
Single-cell RNA sequencing and cell trajectory analysis delineate exocytosis of adipocyte progenitors in mouse subcutaneous white adipose tissue (WAT). Re-analysis performed using publicly available data (GEO dataset # GSM3717977) (Merrick et al., 2019). (**A**) Unsupervised clustering of cells from the subcutaneous WAT confirmed six distinct cell groups represented on a tSNE map. (**B**) Trajectory analyses, color coded based on clustering from panel ‘A’. In similarity to original report (Merrick et al., 2019), *Dpp4*^+^ progenitor cells, *Clec11a*^+^ regulatory cells, and *Dlk1*^+^ pre-adipocytes. Branch identities were confirmed using marker gene expression. (**C**) Pseudotemporal cell ordering of adipocytes along differentiation trajectories. *Clec11a*^+^ regulatory cells (gray) were not included for downstream analyses. (**D**) Pseudotemporal expression heatmap of genes previously linked to activating/promoting exocytosis. Dotted line denotes branch point between mature and regulatory adipocyte populations. (**E**) Combined, normalized expression of pro-exocytosis genes shows enrichment (>1) in early progenitors (green shaded area), while more differentiated cells show below average expression.

**Figure 3—figure supplement 4.**
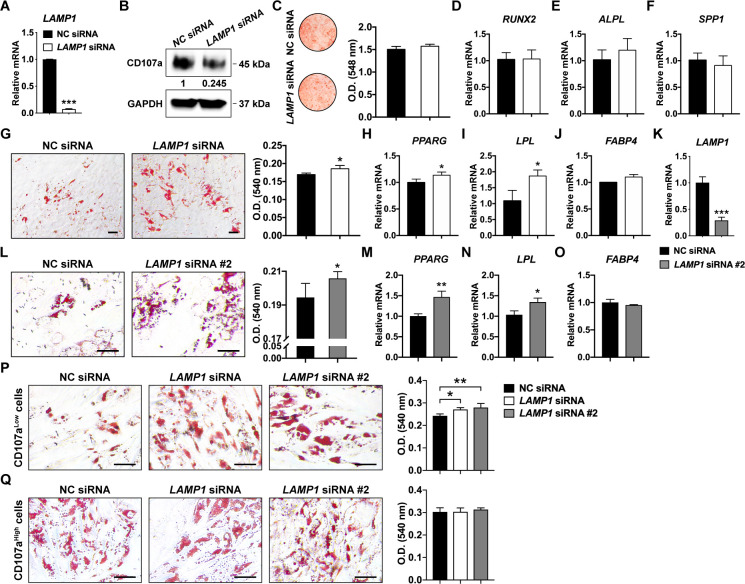
Effect of *LAMP1* knockdown (encoding CD107a) on osteogenic and adipogenic differentiation in human ASCs and CD107a^low/high^ cells. (**A**) Gene expression of *LAMP1* as assessed by qRT-PCR after siRNA-mediated knockdown. (**B**) Western blotting analyses of CD107a expression in human ASCs after siRNA-mediated knockdown. (**C**) Alizarin red (AR) staining (left) and photometric quantification (right) at d 7 of osteogenic differentiation with or without *LAMP1* siRNA. Whole well shown. (**D–F**) Osteogenic gene expression with or without *LAMP1* siRNA assessed by qRT-PCR, including (**D**) *Runt related transcription factor 2* (*RUNX2*), (**E**) *Alkaline phosphatase (ALPL)*, and (**F**) *Osteopontin* (*SPP1*). Gene expression assessed at 7 d of osteogenic differentiation. (**G**) Oil red O (ORO) staining (left) and photometric quantification (right) at d 7 of adipogenic differentiation with or without *LAMP1* siRNA. (**H–J**) Adipogenic gene expression with or without *LAMP1* siRNA, including (**H**) *Peroxisome proliferator-activated receptor-γ* (*PPARG*), (**I**) *Lipoprotein lipase* (*LPL*), and (**J**) *Fatty acid-binding protein 4* (*FABP4*). Gene expression assessed at 7 d of adipogenic differentiation. NC, negative control. (**K**) Gene expression of *LAMP1* as assessed by qRT-PCR after #2 siRNA-mediated knockdown. (**L**) ORO staining (left) and photometric quantification (right) at d 7 of adipogenic differentiation with or without *LAMP1* siRNA #2. (**M–O**) Adipogenic gene expression (7 d) with or without *LAMP1* siRNA #2, including (**M**) *PPARG*, (**N**) *LPL*, and (**O**) *FABP4*. (**P,Q**) ORO staining (left) and photometric quantification (right) at d 7 of adipogenic differentiation with or without *LAMP1* siRNA in (**P**) CD107a^low^ and (**Q**) CD107a^high^ cells. Black scale bar: 50 μm. Data repeated in experimental triplicate wells. *p<0.05; **p<0.01; ***p<0.001. Statistical analysis was performed using a two-tailed Student t-test or a one-way ANOVA followed by Tukey’s post hoc test.

Finally, to confirm that CD107a^low^ and CD107a^high^ mesenchymal cell fractions both represented multipotent precursor cells, parallel chondrogenic differentiation assays were performed in three dimensional micromass culture (Figure 3—figure supplement 1). Here, both cell populations demonstrated a progressive increase in cartilage associated gene expression after 7 d in chondrogenic differentiation conditions. No significant differences in cartilage gene expression were observed between CD107a^low^ and CD107a^high^ cell fractions (Figure 3—figure supplement 1A). Alcian blue stained sections of micromass cultures at 21 d of differentiation likewise showed a similar appearance between CD107a^low^ and CD107a^high^ cells (Figure 3—figure supplement 1B). Thus, CD107a^low^ and CD107a^high^ subpopulations of human SVF both house multipotent mesenchymal cells, but with considerably divergent osteoblastic and adipocytic differentiation potentials.

### CD107a traffics to the cell surface during early adipocyte differentiation

Cell surface expression of CD107a results predominantly from trafficking of endolysosomal CD107a^+^ vesicles to the cell surface. To investigate, unpurified ASCs were exposed to growth conditions or adipogenic differentiation conditions and cell surface CD107a was assessed by immunocytochemistry or flow cytometry (Figure 3H–J). After 3 d exposure to adipogenic conditions, a 12.03 fold increase in immunostaining intensity and a 253.5% increase in the number of CD107a^high^ cells were noted. Flow cytometry across several other human cell types confirmed this finding, including FACS-purified perivascular stem cells (PSCs) (Figure 3K) and culture-defined human bone marrow mesenchymal stem cells (BMSCs) (Figure 3L) (188.1–455.4% increase in CD107a^high^ cell frequency). Parallel experiments were performed under osteogenic differentiation conditions, which found no significant increase in CD107a staining intensity (Figure 3—figure supplement 2). The increase in membranous CD107a during early adipogenesis was reversed by vacuolin-1 (Vac-1), an inhibitor of Ca^2+^-dependent fusion of lysosomes with the cell membrane. Treatment with Vac-1 significantly decreased the frequency of CD107a^high^ ASCs, and prevented an increase of cell surface CD107a by adipogenic conditions (Figure 3M,N).

In order to confirm that exocytosis is a common feature of early adipogenic differentiation, existing single-cell RNA sequencing datasets of human and mouse AT-derived cells were re-assessed (Merrick et al., 2019). Among human AT-derived cells, re-clustering and cell trajectory analysis identified early progenitor cells (expressing *DPP4* and *CD55*), late progenitor cells (expressing *GGT5* and *F3*), as well as an intermediate cell type (middle progenitor cells) (Figure 3O–Q). KEGG terms for exocytosis showed enrichment within early and middle progenitor cells, as visualized by heatmaps across pseudotime (Figure 3R) and normalized expression of overall exocytosis gene activation (Figure 3S). Similar results linking activation of exocytosis to early adipogenic differentiation were obtained from mouse subcutaneous WAT (Figure 3—figure supplement 3; Merrick et al., 2019). Here, after re-clustering and cell trajectory analysis (Figure 3—figure supplement 3A–C), normalized expression of exocytosis gene activation identified similar trends as in human cells (Figure 3—figure supplement 3D,E). Normalized exocytosis gene activation scores were again enriched within early *Dpp4*-expressing progenitor cells in comparison to more mature *Dlk1*-expressing pre-adipocytes.

Knockdown (KD) experiments in human ASCs and CD107a^low/high^ cells did not identify a significant functional role for CD107a in osteo/adipogenesis (Figure 3—figure supplement 4). SiRNA-mediated KD of *LAMP1* (encoding CD107a) showed no appreciable effect on the osteogenic differentiation of human ASCs (Figure 3—figure supplement 4A–F). During adipogenesis, *LAMP1* KD ASCs and CD107a^low^ adventicytes showed a modest increase in lipid droplet accumulation (Figure 3—figure supplement 4G,K,L,P,Q) and likewise a modest increase in expression of adipogenic marker genes (Figure 3—figure supplement 4H–J,M–O). The high adipogenic differentiation potential of CD107a^high^ cells was not significantly altered with *LAMP1* KD (Figure 3—figure supplement 4P,Q). Thus, high expression of membranous CD107a, rather than having vital function in cellular differentiation, correlates with exocytosis during early adipogenic differentiation.

### Transcriptomic analysis suggests a progenitor cell phenotype for CD107a^low^ cells

**Figure 4.**
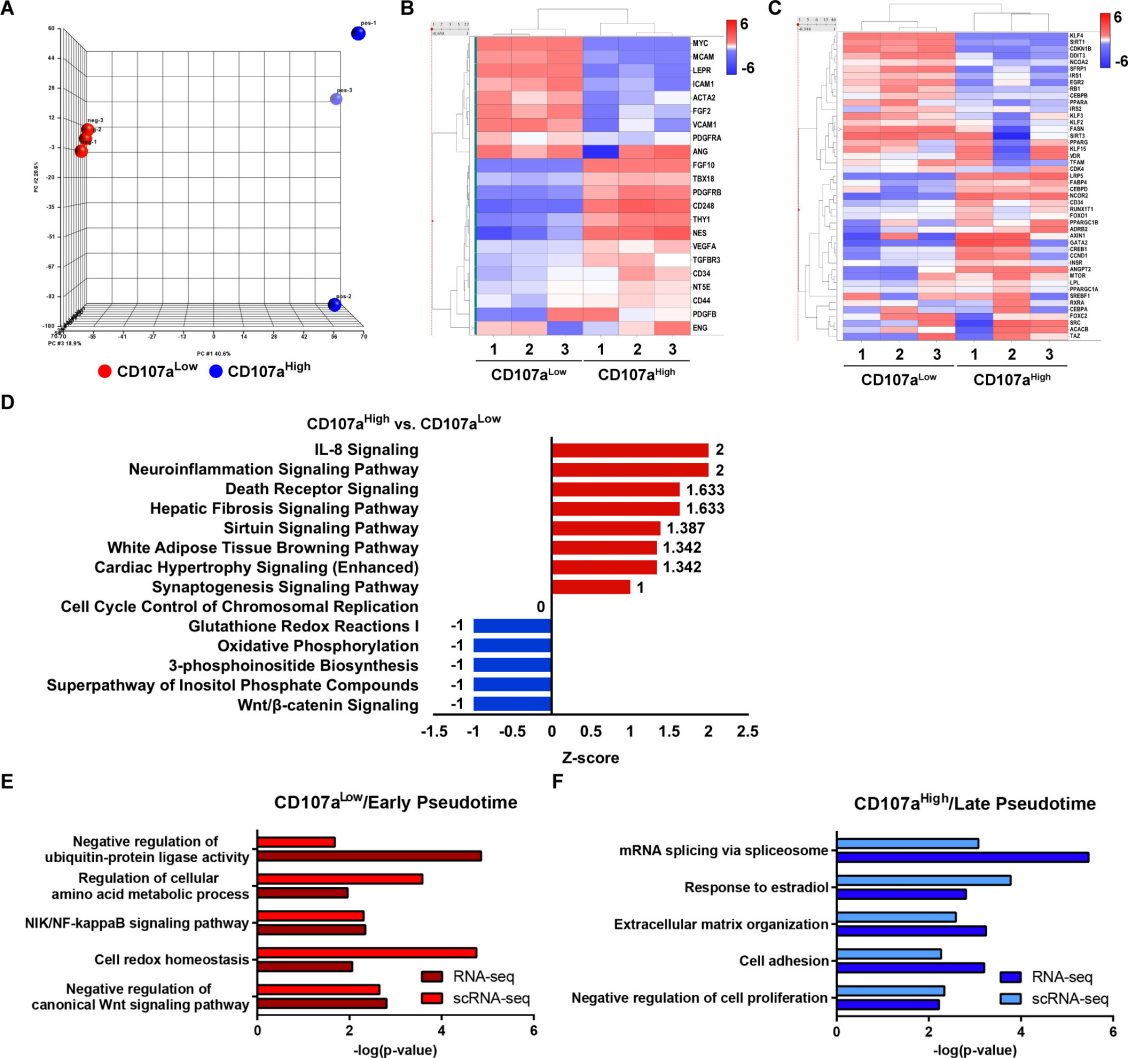
Bulk RNA sequencing among uncultured CD107a^low^ and CD107a^high^ mesenchymal cells and relationship to putative adipose cell hierarchy. (**A–F**) Total RNA sequencing comparison of CD107a^low^CD31^-^CD45^-^ and CD107a^high^CD31^-^CD45^-^ mesenchymal cells from a single human subcutaneous WAT sample. (**A**) Principal component analysis among CD107a^low^CD31^-^CD45^-^ and CD107a^high^CD31^-^CD45^-^ cells. (**B**) Heat map demonstrating mRNA expression levels of stemness-related markers and perivascular cell markers among CD107a^low^CD31^-^CD45^-^ and CD107a^high^CD31^-^CD45^-^ mesenchymal cells. (**C**) Expression of adipogenic gene markers among CD107a^low^CD31^-^CD45^-^ and CD107a^high^CD31^-^CD45^-^ mesenchymal cells, shown in heat map. (**D**) Ingenuity pathway analysis (IPA) identified representative pathways that were upregulated (Z-score >0; red color) or downregulated (Z-score <0; blue color) in CD107a^high^CD31^-^CD45^-^ compared with CD107a^low^CD31^-^CD45^-^ mesenchymal cells. (**E,F**) Comparison of CD107a^high/low^ bulk sequencing data to human SVF single-cell sequencing data (see again Figure 3O–Q). (**E**) Pathways enriched in both CD107a^low^ bulk RNA-seq and early pseudotime genes derived from scRNA-seq. (**F**) Pathways enriched in both CD107a^high^ bulk RNA-seq and late pseudotime genes derived from scRNA-seq.

Differences in differentiation potential were next investigated using transcriptomic analysis of CD107a^low^ and CD107a^high^ stromal cells. RNA sequencing comparative analysis was performed across these two stromal cells. Clear separation between gene expression profiles was observed when comparing CD107a^low^ with CD107a^high^ stromal cells, as assessed by principal component analysis (Figure 4A). Further confirming our FACS purification, endothelial and inflammatory marker genes were rarely or not expressed among CD107a^low^ and CD107a^high^ stromal cells (Figure 4—figure supplement 1). Progenitor cell markers were expressed among both CD107a^low^ and CD107a^high^ stromal cells, with some subtle differences noted (Figure 4B). Transcripts of *MYC, LEPR* (Leptin receptor), *MCAM* (CD146), and *PDGFRA* (Platelet-derived growth factor receptor α) while expressed across all samples were enriched among CD107a^low^ cells. Likewise, *NES* (Nestin), *THY1* (CD90), *PDGFRB* (Platelet-derived growth factor receptor β) and *TBX18* (T-Box transcription factor 18) were expressed across all samples, but more highly among CD107a^high^ cells. Other typical MSC markers were more evenly distributed across cell preparations, including *CD44*, and *NT5E* (CD73). Consistent with in vitro differentiation potential, genes associated with adipogenic differentiation were highly expressed among CD107a^high^ stromal cells, such as *FABP4* (Fatty acid-binding protein 4), *LPL* (Lipoprotein lipase), *PPARGC1A* (PPARG coactivator 1 α), and *CEBPA* (CCAAT enhancer binding protein α). In addition, negative regulators of adipogenesis were increased among CD107a^low^ stromal cells, such as *KLF2* (Krüppel-like factor 2), *KLF3*, *SIRT1* (Sirtuin 1), and *DDIT3* (DNA Damage Inducible Transcript 3) (Figure 4C; Banerjee et al., 2003; Pereira et al., 2004; Sue et al., 2008; Zhou et al., 2015).

**Figure 4—figure supplement 1.**
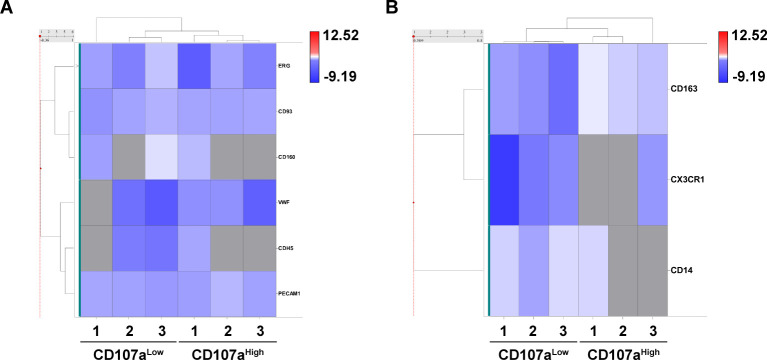
Additional RNA sequencing analysis of CD107a^low^ and CD107a^high^ mesenchymal cells. (**A,B**) Heat map demonstrating mRNA expression levels of endothelial markers (**A**) and inflammatory markers (**B**) among CD107a^low^CD31^-^CD45^-^ and CD107a^high^CD31^-^CD45^-^ mesenchymal cells.

**Figure 4—figure supplement 2.**
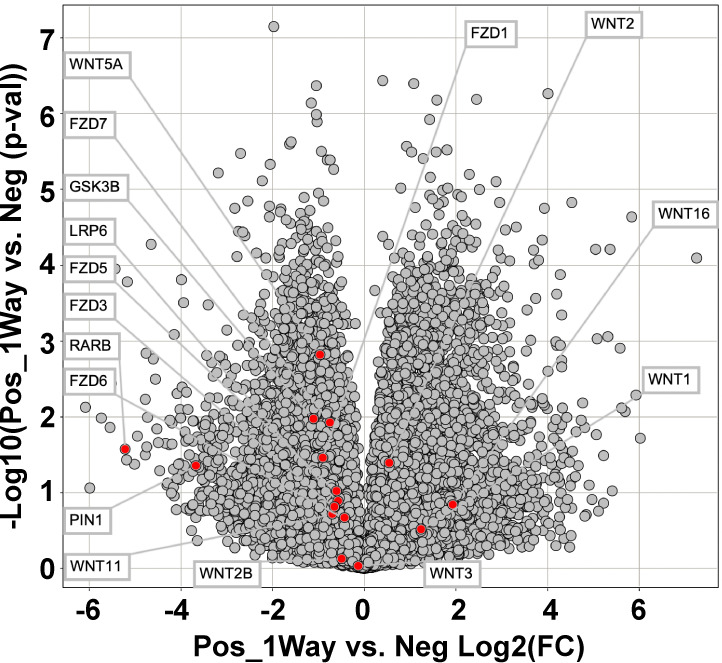
Expression of wnt-related genes among CD107a^low^CD31^-^CD45^-^ and CD107a^high^CD31^-^CD45^-^ mesenchymal cells. Data were shown in volcano plot. All transcripts are shown in light gray, while the transcripts of interest are shown in red.

QIAGEN Ingenuity Pathway Analysis (IPA) showed that the activated pathways in CD107a^high^ stromal cells are associated with the positive regulation of adipogenesis, including, for example, white adipose tissue browning pathway and Sirtuin signaling (Z scores 1.342 and 1.387; CD107a^high^ compared with CD107a^low^ stromal cells; Figure 4D; Kurylowicz, 2019). Conversely, upregulated signaling pathways in CD107a^low^ stromal cells included Wnt/β-catenin signaling as well as pathways associated with cellular respiration and metabolism, including Oxidative Phosphorylation and Glutathione metabolism (Z scores −1; CD107a^high^ compared with CD107a^low^ stromal cells; Figure 4D and Figure 4—figure supplement 2). In order to further evaluate differences in CD107a cell fractions, pathway analyses were next cross-referenced with prior AT-derived single-cell RNA sequencing data (see again Figure 3O). Highly enriched GO terms among CD107a^low^ stromal cells were likewise found to be enriched among *DPP4*^+^ cell fractions during ‘early’ pseudotime (Figure 4E). This included terms associated with Wnt signaling as well as energy metabolism. Conversely, GO terms enriched within CD107a^high^ stromal cells were likewise enriched among *GGT5*^+^ cell fractions within ‘late’ pseudotime (Figure 4F). This included terms associated with the regulation of cellular proliferation, cell adhesion, as well as remodeling of extracellular matrix.

### CD107a^low^ rather than CD107a^high^ cells induce ectopic bone formation

**Figure 5.**
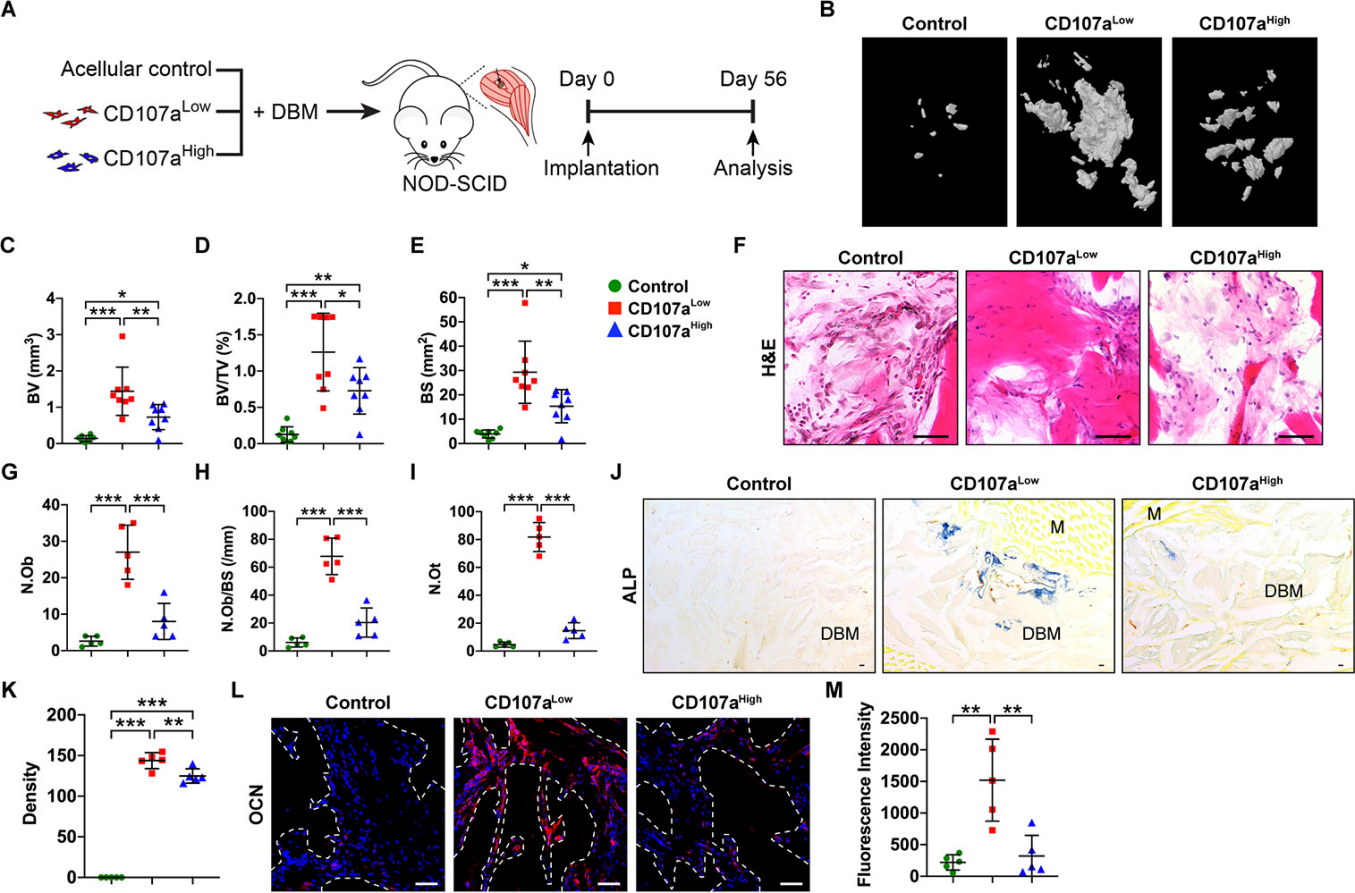
CD107a^low^ mesenchymal cells promote ectopic bone formation in vivo. (**A**) FACS-purified CD107a^low^CD31^-^CD45^-^ and CD107a^high^CD31^-^CD45^-^ mesenchymal cells from the same human subcutaneous WAT sample were implanted intramuscularly in equal numbers in the hindlimbs of NOD-SCID mice. A demineralized bone matrix (DBM) carrier was used, and an acellular control used as a further comparison. Bone formation was assayed after eight wks. Further details on implant composition and animal allocation are found in Supplementary file 5. (**B**) Representative micro-computed tomography (μCT) reconstruction images of the implant site among control (DBM only), CD107a^low^, and CD107a^high^ cell grafts. Mineralized bone appears gray. (**C–E**) μCT based quantification of ectopic bone formation, including (**C**) Bone volume (BV), (**D**) fractional Bone volume (BV/TV), and (**E**) bone surface (BS). (**F**) Representative histologic appearance by routine H and E of the implant sites among control (DBM only), CD107a^low^, and CD107a^high^ cell grafts. (**G–I**) Bone histomorphometric measurements among each treatment group, including (**G**) osteoblast number (N.Ob), (**H**) osteoblast number per bone surface (N.Ob/BS), and (**I**) osteocyte number (N.Ot). (**J,K**) Representative alkaline phosphatase (ALP) staining appearing blue (**J**), and photographic quantification (**K**). (**L,M**) Representative Osteocalcin (OCN) immunohistochemical staining (**L**), and photographic quantification (**M**). OCN immunostaining appears red, while DAPI nuclear counterstain appears blue. Dots in scatterplots represent values from individual implants, while mean and one SD are indicated by crosshairs and whiskers. M, muscle. *p<0.05; **p<0.01; ***p<0.001. Statistical analysis was performed using a one-way ANOVA followed by Tukey’s post hoc test. N = 8 implants per group. Black and white scale bars: 50 μm.

We next sought to extend our findings to xenotransplantation studies. If a CD107a^low^ mesenchymal cell population over-represents stem/osteoblast precursor cells, we hypothesized that CD107a^low^ cells would preferentially form ectopic bone within an intramuscular transplantation model (Figure 5; James et al., 2012c; Meyers et al., 2018b). First, CD107a^low^ and CD107a^high^ cell subsets were derived from the same patient sample and mixed mechanically with a demineralized bone matrix putty carrier (DBX Putty, MTF Biologics) before intramuscular implantation in NOD-SCID mice. The carrier without cells was used as an acellular control. Details of cell implant composition and animal allocation are summarized in Supplementary file 5. Intramuscular implants were imaged by micro-computed tomography (μCT) at 8 weeks, demonstrating an accumulation of bone tissue among CD107a^low^ laden implants in relation to either CD107a^high^ implants or acellular control (Figure 5A). Quantitative μCT analysis demonstrated a significant increase in bone volume (BV, 97.7% increase), fractional bone volume (BV/TV, 73.4% increase), and bone surfaces (BS, 91.4% increase) among CD107a^low^ as compared to CD107a^high^ implants (Figure 5B–D). Albeit to a lesser degree, CD107a^high^ cells did exhibit bone-forming potential in comparison to acellular control (292.1–473.3% increase in μCT quantitative metrics, Figure 5B–D). Histologic analysis revealed conspicuous areas of woven bone among CD107a^low^ laden implants, which were not commonly seen among CD107a^high^ implants (Figure 5E). Bone histomorphometric analysis confirmed these observations, demonstrating significantly increased osteoblast number (N.Ob, 237.5% increase), increased osteoblast number per bone surface (N.Ob/BS, 232.5% increase), and osteocyte number (N.Ot, 460.3% increase) (Figure 5F–H). ALP staining and semi-quantitative analysis confirmed an overall increase in serial sections of CD107a^low^ treated implants (Figure 5I,J, 14.3% increase among CD107a^low^ implant sites). Enrichment in the terminal osteogenic differentiation marker osteocalcin (OCN) was also confirmed among CD107a^low^ implants, shown by immunostaining and semi-quantitative analysis (Figure 5K,L, 345.3% increase among CD107a^low^ implant sites). Detection of human nuclear antigen (HNA) among implant sites confirmed the persistence of human cells across both groups, which were overall similar in frequency (Figure 5—figure supplement 1).

**Figure 5—figure supplement 1.**
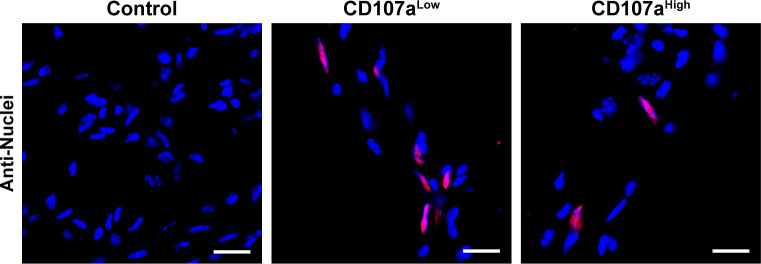
Persistence of human CD107a^low^ and CD107a^high^ cells within intramuscular implants within NOD-SCID mice. Sections were obtained at 8 weeks post-implantation, and immunofluorescence staining of human-specific nuclei performed within CD107a^low^ or CD107a^high^ laden implants, in comparison to acellular control. White scale bar: 20 μm. N = 8 implants analyzed per treatment group.

### CD107a^low^ rather than CD107a^high^ mesenchymal cells induce spine fusion

**Figure 6.**
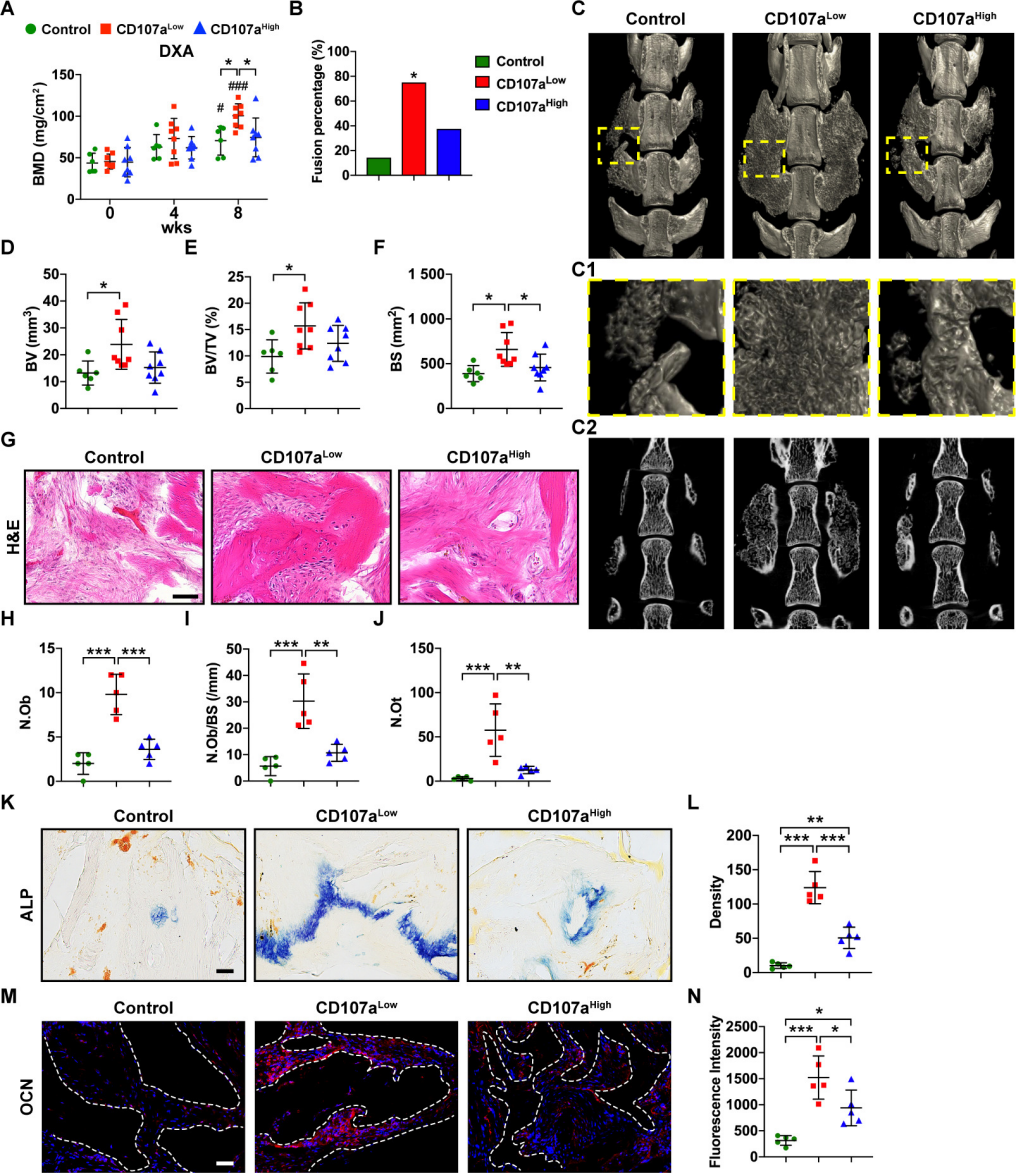
CD107a^low^ mesenchymal cells induce spine fusion in vivo. FACS-purified CD107a^low^CD31^-^CD45^-^ and CD107a^high^CD31^-^CD45^-^ mesenchymal cells from the same human subcutaneous WAT sample were implanted in equal numbers in a posterolateral spinal fusion model in athymic rats. A demineralized bone matrix (DBM) carrier was used, and an acellular control used as a further comparison. Animals were analyzed at up to eight wks post-operatively. (**A**) Bone mineral density (BMD) assessed by DXA (dual-energy X-ray absorptiometry) within the lumbar implantation site, at 0, 4, and 8 wks. (**B**) Spine fusion rate, assessed by manual palpation after eight wks. *: CD107a^low^ compared with acellular control. (**C**) Representative micro-computed tomography (μCT) reconstruction images of the spine fusion site among CD107a^low^ and CD107a^high^ treated samples, in comparison to acellular control. Images are shown from the dorsal aspect. (**C1**) Corresponding high magnification μCT reconstruction of the fusion site. (**C2**) Corresponding coronal μCT cross-sectional image. (**D–F**) μCT-based quantification of bone formation within the spine fusion site, including (**D**) Bone volume (BV), (**E**) fractional Bone volume (BV/TV), and (**F**) bone surface (BS). (**G**) Representative histologic appearance by routine H and E of the implant sites among control (DBM only), CD107a^low^, and CD107a^high^ cell grafts within the spine fusion site. (**H–J**) Bone histomorphometric measurements among each treatment group, including (**H**) osteoblast number (N.Ob), (**I**) osteoblast number per bone surface (N.Ob/BS), and (**J**) osteocyte number (N.Ot). (**K,L**) Representative alkaline phosphatase (ALP) staining appearing blue (**K**), and photographic quantification within the spine fusion site (**L**). (**M**) Representative Osteocalcin (OCN) immunohistochemical staining (**M**), and photographic quantification within the spine fusion site (**N**). OCN immunostaining appears red, while DAPI nuclear counterstain appears blue. Dots in scatterplots represent values from individual animal measurements, while mean and one SD are indicated by crosshairs and whiskers. ^#^p<0.05 and ^###^p<0.001 in relation to corresponding 0 wk timepoint; *p<0.05; **p<0.01; ***p<0.001. Statistical analysis was performed using a two-way ANOVA (**A**) or one-way ANOVA followed by Tukey’s post hoc test (**D–N**). N = 6–8 animals per group. Black and white scale bars: 50 μm.

Having observed that CD107a^low^ cell preparations demonstrate enhanced ectopic bone formation, we next challenged these cells to a posterolateral lumbar spine fusion model within athymic rats (Figure 6; Chung et al., 2014; Lee et al., 2015). CD107a^low^ and CD107a^high^ cell subsets from patient-identical samples were implanted bilaterally in an L4-L5 spine fusion model (Figure 6—figure supplement 1). Details of cell implant composition and animal allocation are summarized in Supplementary file 6. A qualitative increase in radiodensity was observed among CD107a^low^ treated animals within the spine fusion bed over the post-operative period by high-resolution roentgenography (Figure 6—figure supplement 2). Progressive increase in density of the implant sites was confirmed by dual-energy X-ray absorptiometry (DXA)-based quantification, with a gradual and significant increase in CD107a^low^ treated spinal implants in comparison to either CD107a^high^ treated cells or control (Figure 6A). Fusion rate was next assessed by a validated manual palpation scoring (Figure 6B; Grauer et al., 2001). Consistent with prior studies (Chung et al., 2014), after 8 weeks acellular control-treated animals showed 14.3% fusion (1/7 animals). Analyses performed after 8 weeks demonstrated 62.5% spine fusion among CD107a^low^ treated animals (6/8 animals). In comparison, CD107a^high^ treated animals showed 37.5% fusion (3/8 animals). μCT imaging and reconstructions demonstrated lack of bone bridging within the spinal fusion segments of control-treated and CD107a^high^ treated implant sites (Figure 6C). In comparison, more robust evidence of bone bridging was observed among CD107a^low^ spine fusion segments (Figure 6C). Quantitative μCT analysis demonstrated a significant increase in bone volume (BV), fractional bone volume (BV/TV), and bone surfaces (BS) among CD107a^low^ implant sites in comparison to acellular control (Figure 6D–F, 58.6–80.7% increase across μCT metrics). In contrast, CD107a^high^ spine fusion segments demonstrate no statistically significant change in μCT assessments in comparison to acellular control (Figure 6D–F, 15.3–25.2% change in comparison to acellular control). These findings were confirmed using histologic and histomorphometric assessments of the spinal fusion segment across treatment groups (Figure 6G–J). Histologic analysis revealed conspicuous areas of woven bone among CD107a^low^ implants, which were not commonly seen among CD107a^high^ implants (Figure 6G). Bone histomorphometric analysis confirmed these observations, demonstrating significantly increased osteoblast number (172.2% increase among CD107a^low^ implant sites in comparison to CD107a^high^ implant sites), increased osteoblast number per bone surface (183.5% increase), and osteocyte number (357.1% increase) (Figure 6H–J). ALP enzymatic staining and OCN immunohistochemical staining confirmed the above findings (Figure 6K–N). In summary, CD107a^low^ but not CD107a^high^ mesenchymal cell demonstrate improvements in bone-forming potential across two orthopaedic models.

**Figure 6—figure supplement 1.**
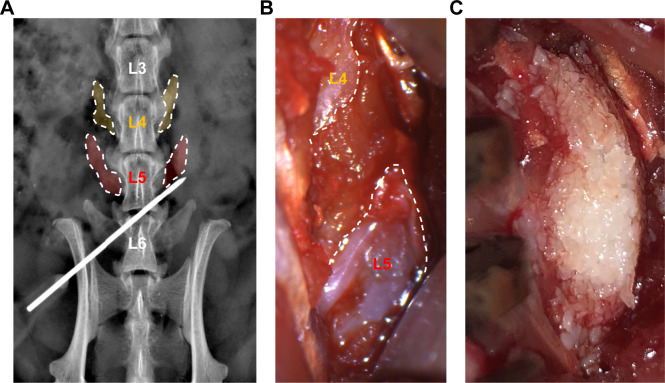
Illustration of procedure for posterolateral lumbar spine fusion in athymic rats. (**A**) Intraoperative postero-anterior high-resolution x-ray to confirm the bony landmarks. The needle is indicating the base of L5 right transverse process. (**B**) Intraoperative microsurgical image of the right paraspinal space after decortication of L5 transverse process. (**C**) Appropriate place and shape of the bone graft substitute in the area encompassing the L4-L5 transverse processes. See Supplementary file 6 for animal allocation.

**Figure 6—figure supplement 2.**
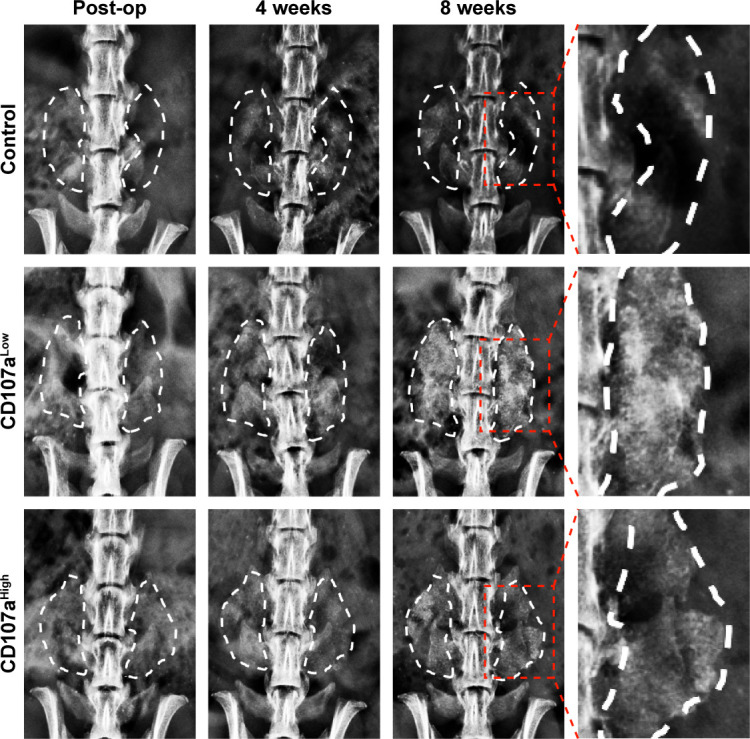
High-resolution roentgenography (XR) demonstrates ossification of spinal fusion implants within CD107a^low^ treated sites. Representative postero-anterior XR images of the lumbar spine post-operatively (far left), 4 weeks (middle left), and 8 weeks (middle and far right) after implantation. See Supplementary file 6 for animal allocation.

## Discussion

Mesenchymal progenitor cells are broadly distributed in post-natal organs, where they are concentrated principally in perivascular areas. Microvascular pericytes were first recognized to include such progenitors since they grow into mesenchymal stem cells in culture (Crisan et al., 2008). The outermost layer enwrapping arteries and veins, or *tunica adventitia*, that used to be considered as a mere fibroblast-populated collagen sheath anchoring vessels within tissues, is also home to presumptive MSCs (Corselli et al., 2012; Kramann et al., 2016). Here, we have used an antibody array targeting all human CD surface markers to identify several novel antigens expressed by human adipose tissue-resident perivascular cells. We found, among other surface antigens, that CD107a, aka LAMP-1, is expressed at the surface of subsets of adventitial cells and pericytes, which was confirmed in terms of gene expression on RNA sequencing libraries, and corroborated by immunohistochemistry, surface CD107a being co-expressed with canonical markers of pericytes and adventitial cells. Further, flow cytometry analysis of the total stromal vascular fraction extracted from adipose tissue showed a continuum of non-endothelial, non-hematopoietic CD107a expressing cells that could be gated back to pericytes and adventitial cells. Altogether, these results confirmed unequivocally that surface CD107a/LAMP-1, used generally as a marker of NK cell activity (Bryceson et al., 2005), is present on subsets of human MSC-related perivascular cells, and established the conditions for the purification of these cells by flow cytometry.

Albeit not described before at the surface of human pericytes and other perivascular cells, CD107a has been earlier detected on human bone marrow and dental conventionally cultured MSCs, where it binds the enamel matrix protein amelogenin and in turn induces cell proliferation (Huang et al., 2010). Besides, CD146^+^CD107a^+^ human bone marrow MSCs have been recently described as endowed with the highest immunomodulatory and secretory, hence therapeutic, potential in experimental joint inflammation (Bowles et al., 2019). Besides suggested association of surface CD107a with progenitor cell proliferation and immunomodulation related tissue repair (Bowles et al., 2019; Huang et al., 2010), lysosomal CD107a has also been linked to neural stem cell potential (Yagi et al., 2010). Here, we show that surface expression of CD107a divides adipocyte- from osteoblast precursors within human perivasculature. Adipocyte progenitor distribution was virtually confined to the CD107a^high^ cell subset; in agreement, adipogenic potential in culture was restricted to this cell compartment. However, knockdown of CD107a in ASCs slightly promoted adipogenic differentiation, suggesting that CD107a can be used for identification of functionally relevant subsets, which is likely not explained by intrinsic function of CD107a protein. CD107a is thought to be responsible for maintaining the structural integrity of the lysosomal compartment (Eskelinen, 2006). Lysosomes provide the degradative enzymes for autophagy and are involved in autophagy regulation, primarily through its relationship with the master kinase complex, mTORC1 (Mrschtik and Ryan, 2015; Yim and Mizushima, 2020). Therefore, CD107a may also play an essential role in autophagy, which itself has been shown crucial for adipocyte differentiation (Guo et al., 2013). Whether CD107a^high^ cells enhance adipogenesis by promoting autophagy is an interesting and worthy follow-up topic. Conversely, osteogenic ability in vitro was almost totally restricted to purified CD107a^low^ cells, which also exhibited the highest CFU-F potential. We confirmed the exclusive bone-forming potential of CD107a^low^ cells in vivo, in situations of ectopic intramuscular ossification and lumbar spine fusion. Thus, CD107a^low^ cells likely represent a more progenitor cell, while CD107a^high^ cells are a more mature, differentiated cell population. Similar results are found in the hematopoietic system, where the most primitive hematopoietic stem cells are Thy-1^low^, whereas Thy-1^high^ cells belong to a well-defined blood cell lineage (Spangrude et al., 1988).

The natural function of these ubiquitous perivascular mesenchymal progenitor cells remains a dominant, yet unanswered question. While subsets of mouse pericytes are known, in contexts of experimental disease or injury, to give rise in situ to white adipocytes, follicular dendritic cells, myoblasts, and myofibroblasts (Dellavalle et al., 2011; Dulauroy et al., 2012; Krautler et al., 2012; Murray et al., 2017; Tang et al., 2008), the osteogenic and chondrogenic potentials present in these microvascular cells are unlikely to be ever used in the turnover and repair of soft tissues. On the other hand, a contribution to blood vessel pathologic remodeling of perivascular presumptive MSCs has been recognized, as osteoblast and smooth muscle cell forerunners for calcific arteriosclerosis and atherosclerosis, respectively (Kramann et al., 2016). How such pathogenic side effects exerted by perivascular progenitors are counterbalanced by beneficial contributions to tissue homeostasis is not known, but might be related to differential recruitment of uni- or multipotent progenitors, as well as activation in these cells of mechanisms independent of cell differentiation such as growth factor production or cell contact-mediated immunomodulation (Pittenger et al., 2019). A precise phenotypic and functional characterization of perivascular progenitor cells is a requisite for the understanding of the role of these cells in developmental, regenerative, and pathophysiologic processes. In particular, unwrapping the intrinsic heterogeneity of these cells is a prioritized, ongoing process.

Although abundant evidence of the osteogenic potential of perivascular cells exists in humans and mice (Askarinam et al., 2013; Chung et al., 2014; Corselli et al., 2012; Crisan et al., 2008; James et al., 2017; James and Péault, 2019; James et al., 2012a; James et al., 2012a; James et al., 2012b; James et al., 2012c; Kramann et al., 2016; Lee et al., 2015; Meyers et al., 2018b; Meyers et al., 2018b; Tawonsawatruk et al., 2016; Wang et al., 2020; Xu et al., 2019), there is recent proof that this competence is restricted to discrete cell subsets. For instance, CD10 expression marks a subset of human adipose tissue adventitial cells with higher bone-forming potential (Ding et al., 2019), and the highest calcification potential is attributed to mouse adventitial cells co-expressing CD34 and PDGFRα (Wang et al., 2020). On the other hand, human perivascular cells expressing the ROR2 Wnt receptor exhibit stronger chondrogenic ability than ROR2 negative counterparts (Dickinson et al., 2017). Therefore, the concept is emerging of a developmental micro-heterogeneity of perivascular cells, the surrounding niches being suggested to host a whole hierarchy of mesenchymal progenitor cells already documented to include adipocyte-, chondrocyte-, and osteoblast progenitors. Although the data herein were obtained on abdominal subcutaneous adipose tissue, our preliminary results indicate that the same cell partition can be achieved in other fat depots. Moreover, we have observed the same restriction of adipogenic and osteogenic potentials, respectively, to CD107a^high^ and CD107a^low^ perivascular cells sorted from the human placenta, which suggests the broad and possibly ubiquitous distribution of these functionally distinct subpopulations. Strikingly, these tissues are not natural sites of ossification, which raises the recurring question of the physiologic significance of these unrelated developmental potentials. It is conceivable that mesodermal cell turnover and regeneration in adult tissues be mediated exclusively by multipotent, MSC like cells, irrelevant potentials in a given tissue being always repressed. It is more difficult to justify that progenitors committed to a given cell compartment be maintained in a tissue devoid of this very cell lineage, such as osteogenic dedicated progenitors in adipose tissue, at the expense of adipogenic cells. Since the hypothesis that these unrelated progenitors can be mobilized through blood circulation to drive regeneration in other organs is not supported for the moment, we can only speculate that such atypical differentiation potentials are irreversibly associated with other tissue repair mechanisms of broader applicability, which remain to be identified. Further characterization of the role of CD107a at the cell surface may contribute to clarifying this issue.

Our in vitro studies, coupled with single-cell transcriptomics, suggest that CD107a, an endolysosome transmembrane protein, traffics to the cell surface during early adipogenesis, suggesting a specific function in this cell lineage. Future studies will tell whether CD107a/LAMP-1, a unique novel marker of the perivascular mesenchymal stem cell hierarchy, will also shed light on the tissue regeneration mechanisms initiated in this niche.

## Materials and methods

### Immunohistochemistry and microscopy

All human tissues were obtained under Johns Hopkins University institutional IRB approval with a waiver of informed consent. For histology, human subcutaneous fat tissue was obtained from three anonymized female donors from the abdominal or thigh area. Human fat tissue was embedded in optimal cutting temperature compound (OCT) (Sakura, Torrance, CA), and cryo-sectioned at 30 μm thickness. For immunofluorescent staining, all sections were blocked with 5% goat serum in PBS for 1 hr at room temperature (RT). The following primary antibodies were used: anti-αSMA (RRID:AB_1951138, 1:100), anti-CD107a (RRID:AB_1727417/RRID:AB_10719137/RRID:AB_470708/RRID:AB_449893, 1:100), anti-CD31 (RRID:AB_448167/RRID:AB_726362, 1:100), anti-CD34 (RRID:AB_1640331, 1:100), anti-CD146 (RRID:AB_2143375, 1:100), or anti-Gli1 (RRID:AB_880198, 1:100; see antibody details in Supplementary file 7) for overnight incubation at 4^o^ C. Next, anti-rabbit Alexa Fluor 647-conjugated (RRID:AB_2722623, 1:200), anti-mouse Alexa Fluor 488-conjugated (RRID:AB_2688012, 1:200), anti-goat Alexa Fluor 647-conjugated (RRID:AB_2687955, 1:200), or anti-rat Alexa Fluor 647-conjugated secondary antibodies (RRID:AB_2864291, 1:200, Abcam, San Francisco, CA) were used (incubation 2 hr at RT). DAPI mounting medium was used (RRID:AB_2336788, Vector laboratories, Burlingame, CA), and visualized using a Zeiss 800 confocal microscope (Zeiss, Thornwood, NY). For colorimetric immunohistochemistry staining, sections were blocked with 2.5% horse serum for 20 min at RT. Anti-CD107a primary antibody (RRID:AB_470708, Abcam, 1:100) was added and incubated overnight at 4° C. Next, incubation with alkaline phosphatase (AP) polymer anti-mouse IgG reagent was performed for 30 min (RRID:AB_2336535, MP-5402, Vector laboratories), followed by AP substrate solution (RRID:AB_2336847, SK-5100, Vector laboratories), followed by hematoxylin counterstain and microscopic imaging using a Leica DM6 B microscope (Leica Microsystems Inc, Wetzlar, Germany).

### Adipose-derived stromal cells (ASCs) isolation and FACS isolation of human AT cell populations

For cell isolation, human lipoaspirate was obtained from healthy adult donors and was stored no more than 72 hr at 4°C before processing with some modifications from prior protocols (Wang et al., 2019; Xu et al., 2019). Patient gender and donor area are shown in Supplementary file 1. Equal volume of phosphate-buffered saline (PBS) was used to wash lipoaspirates. The washed lipoaspirate was digested with 1 mg/ml type II collagenase (Washington Biochemical; Lakewood, NJ) in Dulbecco's Modified Eagle's Medium (DMEM) containing 0.5% bovine serum albumin (Sigma-Aldrich, St. Louis, MO) at 37°C for 60 min under agitation, followed by centrifugation to remove adipocytes. The cell pellet was resuspended and incubated in red cell lysis buffer (155 mM NH_4_Cl, 10 mM KHCO3, and 0.1 mM EDTA) at RT for 10 min. After centrifugation, the stromal vascular fraction (SVF) was resuspended in PBS and filtered at 40 μm. In select studies, SVF was culture- expanded as adipose-derived stromal cells (ASCs) for further evaluation. Human ASCs were cultured and used for experiments at passage 2–6. Fluorescence activated cell sorting (FACS) was next performed using a Beckman MoFlo (Beckman, Indianapolis, IN), with analysis performed using the FlowJo software (RRID:SCR_008520, BD Biosciences, San Jose, CA). Cells were incubated with anti-CD45-allophycocyanin-cyanin 7 (RRID:AB_396891, 1:30; BD Pharmingen, San Diego, CA), anti-CD31-allophycocyanin-cyanin 7 (RRID:AB_2738350, 1:100, BD Pharmingen), anti-CD34 (RRID:AB_11154586, 1:60, BD Pharmingen), anti-CD146 (RRID:AB_324069, 1:100, Bio-Rad, Hercules, CA), and/or anti-CD107a-allophycocyanin (RRID:AB_1727417, 1:20; BD Pharmingen), for 20 min on ice. Propidium iodide (PI) staining solution (BD Pharmingen) was used to gate out non-viable cells. See Supplementary file 7 for a list of antibodies used. Gating was performed to isolate either CD146^+^ pericytes (CD146^+^CD34^-^CD31^-^CD45^-^), CD34^+^ adventicytes (CD34^+^CD146^-^CD31^-^CD45^-^), CD107a^low^ cells (CD107a^low^CD31^-^CD45^-^) or CD107a^high^ cells (CD107a^high^CD31^-^CD45^-^). Cell surface markers were analyzed using either Lyoplate (BD Biosciences) or flow cytometry. For flow cytometry, cells were incubated with the following antibodies for 20 min on ice: anti-CD34 PE-CF594 (RRID:AB_11154586), anti-CD146 FITC (RRID:AB_324069), anti-CD44 Alexa Fluor 700 (RRID:AB_10645788), anti-CD73 PE (RRID:AB_2033967), anti-CD90 FITC (RRID:AB_395969), anti-CD105 PE-CF594 (RRID:AB_11154054), and anti-CD107a APC (RRID:AB_1727417, Supplementary file 7). For select studies using culture-expanded cells, flow cytometry was performed after trypsinization and cell re-suspension in HBSS (Life Technologies, Gaithersburg, MD) with 0.5% bovine serum albumin (Sigma-Aldrich). All cells were cultured at 37°C in a humidified atmosphere containing 95% air and 5% CO_2_. Unless otherwise stated, cells were cultured in Endothelial Cell Growth Medium-2 (EGM-2; Lonza, Gaithersburg, MD).

### Human bone marrow mesenchymal stem cell isolation

Bone marrow mesenchymal stem cells (BMSCs) from anonymized human femur and tibia were isolated using previously reported methods (Xu et al., 2019). Marrow cells were flushed with PBS and passed through a 70 μm cell strainer (BD Biosciences) to obtain single cells, which were seeded into T75 flasks. Non-adherent cells were removed after 5 d and medium was changed every 3 d. BMSCs were cultured in growth medium consisting of DMEM, 15% fetal bovine serum (FBS; Gibco, Grand Island, NY), 1% penicillin/streptomycin (Gibco).

### Identification of novel human perivascular cell markers using lyoplate

The BD Lyoplate Human Cell Surface Marker Screening Panel contains 242 purified and lyophilized monoclonal antibodies to cell surface markers, along with AlexaFluor 647 conjugated goat anti-mouse Ig and goat anti-rat Ig secondary antibodies, distributed in three 96-well plates, as well as mouse and rat isotype controls for assessing isotype-specific background. The Lyoplate array was used according to manufacturer’s instructions. Aspirated human subcutaneous fat was digested with collagenase, washed by centrifugation and the SVF recovered as described above. After washing and red cell lysis, SVF cells were stained with the following reagents: DAPI 1/100 (1 μg/ml final), FITC-CD146 1/40, PE-CD45 1/20, PE-Cy7-CD34 1/33. Using a multi-channel pipette, 100 μl aliquots of antibody stained SVF (500,000 to 1 million cells) were distributed in the wells, and 50 μl of reconstituted Lyoplate antibody solution were added to each well according to the template. Plates were incubated on ice in the dark for 30 min, then cells were washed twice by adding 100 μl of staining solution to each well and spinning at 300xg for 5 min. 100 μl of 4% paraformaldehyde (PFA) were added to each well and incubated at RT for 30 min. Labeled cells were washed again and either stored at 4°C or analyzed directly on a LSR II flow cytometer (BD Biosciences).

### Transcriptomics

In select experiments, global gene expression analysis of CD107a^low^CD31^-^CD45^-^ and CD107a^high^CD31^-^CD45^-^ cells from adipose tissue was performed. The RNA content of CD107a^low^ and CD107a^high^ cells was detected by total RNA sequencing. Briefly, total RNA was extracted from CD107a^low^ and CD107a^high^ cells with Trizol (Life Technologies). After purification and reverse transcription, cDNA samples were sent to the JHMI Transcriptomics and Deep Sequencing Core and quantified by deep sequencing with the Illumina NextSeq 500 platform (Illumina, San Diego, CA). Data were analyzed using software packages including Partek Genomics Suite (RRID:SCR_011860), Spotfire DecisionSite with Functional Genomics (RRID:SCR_008858), and QIAGEN Ingenuity Pathway Analysis (RRID:SCR_008653).

### Single-cell RNA sequencing (scRNA-seq)

ScRNA-seq data were obtained from the Gene Expression Omnibus (GEO) repository, accession number GSE128889 (GSM3717979, GSM3717977). Initial quality control removed cells expressing >200 and<6000 genes and a mitochondrial content >5%. Data normalization, dimensional reduction and clustering were conducted in Seurat (RRID:SCR_016341) as previously described in the original publication with the exception of altered clustering resolutions. Trajectory plots were generated in Monocle (RRID:SCR_018685) as previously described. For exocytosis pathway activation, a gene list of exocytosis activating/promoting genes was generated from the previously annotated KEGG pathway. Gene lists were filtered for genes that met the Monocle cutoff criteria as an expressed gene (expressed in a minimum of 10 cells). Expression levels were normalized first for individual cell UMI counts, then to the average gene expression across the whole sample population. This normalized expression across pseudotime was displayed following average with nearest neighbors. Values above one indicate enriched expression of genes associated with exocytosis while values below one indicate expression below population averages. Pathway analyses were conducted on CD107a^low^ and CD107a^high^ cells from bulk RNA-seq experiments, as well as on early- and late-expressing, pseudotemporally-regulated genes derived from scRNA-seq data.

### Osteogenic differentiation assay and ALP/Alizarin red staining

For osteogenic differentiation, cells were seeded at the density of 2.5 × 10^5^/ml in 12- or 24-well plates. Upon confluency, medium was changed to osteogenic differentiation medium, composed of DMEM, 10% FBS (Gibco), 1% penicillin/streptomycin, with 50 μM ascorbic acid, 10 mM β-glycerophosphate, and 100 nM dexamethasone (Sigma-Aldrich) (Xu et al., 2019). Medium was changed every 3 d. Leukocyte Alkaline Phosphatase Kit (Sigma-Aldrich) was used for alkaline phosphatase staining at 3 or 7 d of differentiation. Image J (RRID:SCR_003070) was used to detect the integrated density of ALP staining. Alizarin red S (Sigma-Aldrich) was used to stain cultures at 7 or 10 d of differentiation to detect mineralization. Next, calcium precipitate was dissolved with 0.1N sodium hydroxide and quantified by absorbance at 548 nm. Experiments were performed in N = 3 biological replicates, and in experimental triplicates in each case.

### Adipogenic differentiation assay and oil red O staining

For adipogenic differentiation, cells were seeded at the density of 2.5 × 10^5^/ml in 12- or 24-well plates. Upon subconfluency, adipogenic differentiation medium (DMEM, 1% penicillin/streptomycin, 10% FBS with 1 μM dexamethasone, 200 μM indomethacin, 10 μg/ml insulin, and 500 μM 3-isobutyl-1-methylxanthine (Sigma-Aldrich)) was used. Medium was changed every three d. Cells were fixed with 4% PFA and stained with Oil Red O solution at 5 to 7 d of differentiation (Meyers et al., 2018a). Experiments were performed in N = 3 biological replicates, and in experimental triplicates in each case.

### Chondrogenic differentiation assay and Alcian blue staining

For chondrogenic differentiation, cells were seeded at high-density micromass environment (1 × 10^7^/ml, 10 μl/drop) in 12-well plates and cultured in 37°C. After 4 hr, chondrogenic differentiation medium (DMEM, 1% penicillin/streptomycin, 10% FBS with 10 ng/ml transforming growth factor-β3 (R and D Systems, Minneapolis, MN), 100x ITS+ Premix (Corning Incorporated, Corning, NY), 50 μg/ml ascorbic acid, 40 μg/ml proline, 100 μg/ml pyruvate, and 100 nM dexamethasone (Sigma-Aldrich)) was added. Medium was changed every three d. Cells were fixed with 4% PFA and embedded in OCT for cryosectioning at 18 μm thickness. Slides were stained with Alcian Blue and Fast Red.

### Proliferation assay

Proliferation assays were performed in 96 well plates (2 × 10^3^ cells/well) and measured at 72 hr using the CellTiter96 AQueous One Solution Cell Proliferation Assay kit (MTS, G358A; Promega, Madison, WI). Briefly, 20 μl of MTS solution was added to each well and incubated for 1 hr at 37°C. The absorbance was assayed at 490 nm using Epoch microspectrophotometer (Bio-Tek, Winooski, VT).

### CFU assay

For all CFU assays, 1,000 cells / well were seeded in 6-well plates. For CFU-F analysis, cells were cultivated for 14 d in growth medium, fixed with 100% methanol and stained with 0.5% crystal violet. For CFU-OB assays, cells were cultivated for 7 d in growth medium, followed by 3 d culture in osteogenic differentiation medium followed by alkaline phosphatase staining. For CFU-AD assays, cells were cultured for 8 d in growth medium, followed by 8 d in adipogenic differentiation medium, followed by Oil red O staining. For quantification, the total number of positive colonies was calculated per well. All CFU assays were performed with N = 6 wells per group.

### Immunocytochemistry

Cells were seeded at the density of 1.5 × 10^5^/ml in EZ SLIDE (Merck Millipore, Billerica, MA). After confluence, medium was changed to osteogenic differentiation medium or adipogenic differentiation medium for 3 d. To visualize the membranous expression of CD107a, cells were directly stained with anti-CD107a-allophycocyanin (RRID:AB_1727417, 1:20; BD Pharmingen) for 20 min at 4℃. Then cells were washed with PBS and fixed with 4% PFA, followed by DAPI mounting medium (RRID:AB_2336788, Vector laboratories).

### Vacuolin-1 treatment

To inhibit exocytosis, cells were pre-treated with vacuolin-1 (1 μM; Sigma-Aldrich) for 24 hr before adipogenic differentiation medium was added. Cells were incubated in the presence or absence of vacuolin-1 (1 μM) in adipogenic differentiation medium for 3 d. Surface CD107a expression was detected by flow cytometry or confocal microscopy (Zeiss 800). For immunofluorescence staining, the cell membrane was labeled using Wheat Germ Agglutinin Conjugates (Thermo Fisher Scientific, Waltham, MA).

### siRNA knockdown

In select experiments, siRNA-mediated knockdown of *LAMP1* was performed among primary human ASCs prior to osteogenic or adipogenic differentiation. *LAMP1* siRNA (Cat# s8080 and s8082) and negative control siRNA (Cat# 4390843) were obtained from Thermo Fisher Scientific. TransIT-LT1 Transfection Reagent (Mirus Bio, Madison, WI) was used as described by the manufacturer. The medium was changed after 4 hr. Validation by qRT-PCR was performed.

### Real-time polymerase chain reaction

Gene expression was analyzed with quantitative real-time polymerase chain reaction (qRT-PCR) (Xu et al., 2019). Total RNA was isolated from cells using TRIzol (Life Technologies). Next, RNA was reverse transcribed into cDNA by iScript cDNA Synthesis Kit (Bio-Rad) following manufacturer's instructions. SYBR Green PCR Master Mix (Life Technologies) was used for RT-PCR. See Supplementary file 8 for primer information. N = 3 wells per group were used, with all studies performed in biologic triplicates.

### Western blot

Proteins were extracted from cultured cells following lysis in ice cold RIPA buffer (Thermo Scientific) with protease inhibitor cocktail (Cell Signaling Technology, Danvers, MA, USA). Proteins were separated by SDS–polyacrylamide gel electrophoresis and transferred onto a nitrocellulose membrane. The blotted nitrocellulose membranes were blocked with 5% bovine serum albumin for 1 hr and then probed with primary antibodies at 4°C overnight. Finally, membranes were incubated with a horseradish-peroxidase (HRP)-conjugated secondary antibody and detected by ChemiDoc XRS+ System (Bio-rad). Quantification of protein bands was performed using Image J software (RRID:SCR_003070).

### Intramuscular implantation

Animals were housed and experiments were performed in accordance with institutional guidelines at Johns Hopkins University under ACUC approval. A DBX putty (courtesy of Musculoskeletal Transplant Foundation, Edison, NJ) was used for ectopic bone formation in mice. Briefly, CD107a^low^ or CD107a^high^ cells derived from the same human WAT sample at passage five were prepared at a density of 3 million total cells in 40 μl PBS and mechanically mixed with 45 mg DBX putty. DBX alone was used as an acellular control. The cell preparation was then implanted intramuscularly into the thigh muscle pouch of 8-week-old male NOD-SCID mice (RRID:IMSR_JAX:001303, The Jackson Laboratory, Bar Harbor, ME) as previously described with some modifications (James et al., 2012c). Mice were anesthetized by isoflurane inhalation and premedicated with buprenorphine. Incisions in the hindlimbs were made, and pockets were cut in the biceps femoris muscles by blunt dissection, parallel to the muscle fiber long axis. Dissection methods and the surgical manipulation of tissues were kept as constant as possible across animals. The muscle and skin were each closed with 4–0 Vicryl*Plus sutures (Ethicon Endo-Surgery, Blue Ash, OH). See Supplementary file 5 for an outline of animals per experimental group. Surgical implantations and subsequent analyses were performed blinded to treatment group.

### Lumbar spine fusion

Posterolateral lumbar spinal fusion was performed on 23-week-old male athymic rats (RRID:RGD_2312499) as previously described (Chung et al., 2014). Posterior midline incisions were made over the caudal portion of the lumbar spine, and two separate fascial incisions were made 4 mm bilaterally from the midline. L4 and L5 lumbar spines were exposed by blunt muscle splitting technique and decorticated using a low speed burr and micro-drill (Roboz Surgical Instrument Co., Gaithersburg, MD). Next, DBX (300 μl per side) mixed with CD107a^low^ or CD107a^high^ cells from the same AT sample (1.5 million cells, P3-5 passage) or DBX alone were implanted between the transverse processes bilaterally into the paraspinal muscle bed. Finally, the fasciae and skin were each closed using continuous suture (4–0 Vicryl*Plus, Ethicon Endo-Surgery). In vivo imaging was performed by a combination X-ray/DXA (Faxitron Bioptics, Tucson, AZ) at 0, 4, and 8 weeks after surgery. Rats were sacrificed 8 weeks after surgery, and the spines were harvested for further analysis. See Supplementary file 6 for animals per experimental group. Surgical procedures and subsequent analyses were performed blinded to treatment group.

### Post mortem analyses

Samples were fixed in 4% PFA for 24–48 hr and evaluated using a high-resolution micro-computed tomography (μCT) imaging system (SkyScan 1275; Bruker MicroCT N.V, Kontich, Belgium). For intramuscular implants, scans were obtained at an image resolution of 15 μm with a 1 mm of aluminum filter (X-ray voltage of 65 kVP, anode current of 153 uA, exposure time of 218 ms). For spine fusion samples, scans were obtained at an image resolution of 22 μm with a 1 mm of aluminum filter (X-ray voltage of 55 kVP, anode current of 181 uA, exposure time of 218 ms). NRecon software (SkyScan, Bruker) was used to reconstruct images from the 2D X-ray projections. For the 3D morphometric analyses of images, CTVox and CTAn were used (SkyScan, Bruker). For muscle pouch implantation and spine fusion analysis, volumes of interest were shaped to encompass all the implant and exclude native bone, with a threshold value of 65.

After radiographic imaging, samples were transferred to 14% EDTA for decalcification for 28–60 d. Samples were then embedded in OCT for cryosectioning at 18 μm thickness. H and E and ALP staining were performed on serial sections (Leukocyte Alkaline Phosphatase Kit, Sigma-Aldrich). For immunofluorescent staining, sections were washed with PBS three times and blocked with 5% goat serum in PBS for 1 hr at 25° C. Antigen retrieval was performed by trypsin enzymatic antigen retrieval solution (ab970; Abcam) for 10 min at 25° C. Primary anti-osteocalcin antibody (RRID:AB_10675660, 1:100, Abcam) was added to each section and incubated at 4° C overnight. Next, an Alexa Fluor 647 goat anti-rabbit IgG (H+L) polyclonal (RRID:AB_2722623, 1:200, Abcam) was used as the secondary antibody. Sections were counterstained with DAPI mounting medium (RRID:AB_2336788, Vector laboratories).

### Statistical analysis

Quantitative data are expressed as mean ±one SD. Statistical analyses were performed using the SPSS16.0 software (RRID:SCR_002865) or GraphPad Prism (RRID:SCR_002798, Version 7.0). Our in vitro studies comparing CD107a^low^ to CD107a^high^ cells resulted in effect size of 3.75. Based on α = 0.05 and power = 0.8, statistical significance should be observed with N = 6 animals per group assuming a one-way ANOVA with a 0.05 significance level. Student’s t-test was used for two-group comparisons, and one-way ANOVA test was used for comparisons of three or more groups, followed by Tukey’s post hoc test. Two-way ANOVA test was used for comparisons of two or three groups with different time points. Differences were considered significant with *p<0.05, **p<0.01, and ***p<0.001.

## Data Availability

Expression data that support the findings of this study have been deposited in Gene Expression Omnibus (GEO) with the accession codes GSE148519 and GSE128889 (GSM3717979, GSM3717977). The following dataset was generated: JamesAWXuJ2020Expression data of CD107aLow and CD107aHigh cells isolated from human adipose tissueNCBI Gene Expression OmnibusGSE148519 The following previously published dataset was used: SealePMerrickDSakersA2019Identification of a mesenchymal progenitor cell hierarchy in adipose tissueNCBI Gene Expression OmnibusGSE12888910.1126/science.aav2501PMC681623831023895

## Additional files

Supplementary file 1. Frequency of CD107a^low^CD31^-^CD45^-^ or CD107a^high^CD31^-^CD45^-^ cells by FACS sorting. Percentages are based on cell frequency within the PI- cell population.

Supplementary file 2. CD34 frequency among freshly isolated CD107a^low^ and CD107a^high^ cells.

Supplementary file 3. CD146 frequency among freshly isolated CD107a^low^ and CD107a^high^ cells.

Supplementary file 4. Canonical mesenchymal stem cell (MSC) markers among freshly isolated CD107a^low^ and CD107a^high^ cells.

Supplementary file 5. Animal allocation for intramuscular implantation using 12 NOD-SCID mice. Implants were placed bilaterally, with each animal receiving the same treatment on either hindlimb.

Supplementary file 6. Animal allocation for posterolateral lumbar spine fusion model in athymic rats. Cell-augmented grafts were placed bilaterally on either side of the lumbar spine, with scaffold and cell numbers per side shown.

Supplementary file 7. Antibodies used.

Supplementary file 8. Primers used.

Supplementary file 9. Uncropped versions of representative western blot images from Figure 3—figure supplement 4B.

Transparent reporting form

## Appendix 1

**Appendix 1—key resources table:**

Reagent type (species) or resource	Designation	Source or reference	Identifiers	Additional information
Strain, strain background (mouse)	NOD-SCID	Jackson Laboratory	Strain #001303, RRID:IMSR_JAX:001303	Male, 8 week old
Strain, strain background (rat)	RNU Nude Rat	Charles River Laboratories Inc	Strain #316, RRID:RGD_2312499	Male, 23 week old
Transfected construct (human)	Negative Control siRNA	ThermoFisher Scientific	Cat#4390843	
Transfected construct (human)	LAMP1 siRNA	ThermoFisher Scientific	Cat#4392420 Assay ID s8082	
Transfected construct (human)	LAMP1 siRNA 2#	ThermoFisher Scientific	Cat#4392420 Assay ID s8080	
Antibody	anti-Human CD31-APC-Cy7 (Mouse monoclonal)	BD Pharmingen	Cat# 563653, RRID:AB_2738350	FACS/Flow cytometry (1:100)
Antibody	anti-Human CD31 (Mouse monoclonal)	Abcam	Cat# ab24590, RRID:AB_448167	Immunofluorescent staining (1:100)
Antibody	anti-Human CD31 (Rabbit polyclonal)	Abcam	Cat# ab28364, RRID:AB_726362	Immunofluorescent staining (1:100)
Antibody	anti-Human CD34-PE-CF594 (Mouse monoclonal)	BD Pharmingen	Cat# 562383, RRID:AB_11154586	Flow cytometry (1:60)
Antibody	anti-Human CD34 (Rabbit monoclonal)	Abcam	Cat# ab81289, RRID:AB_1640331	Immunofluorescent staining (1:100)
Antibody	anti-Human CD44-AF700 (Mouse monoclonal)	BD Pharmingen	Cat# 561289, RRID:AB_10645788	Flow cytometry (1:20)
Antibody	anti-Human CD45-APC-Cy7 (Mouse monoclonal)	BD Pharmingen	Cat# 557833, RRID:AB_396891	FACS/Flow cytometry (1:30)
Antibody	anti-Human CD73-PE (Mouse monoclonal)	BD Pharmingen	Cat# 561014, RRID:AB_2033967	Flow cytometry (1:5)
Antibody	anti-Human CD90-FITC (Mouse monoclonal)	BD Pharmingen	Cat# 555595, RRID:AB_395969	Flow cytometry (1:20)
Antibody	anti-Human CD105-PE-CF594 (Mouse monoclonal)	BD Pharmingen	Cat# 562380, RRID:AB_11154054	Flow cytometry (1:20)
Antibody	anti-Human CD107a-APC (Mouse monoclonal)	BD Pharmingen	Cat# 560664, RRID:AB_1727417	FACS/Flow cytometry (1:20)/Immunocytochemistry (1:100)
Antibody	anti-Human CD107a (Mouse monoclonal)	R and D Systems	Cat# MAB4800, RRID:AB_10719137	Immunofluorescent staining (1:100)
Antibody	anti-Human CD107a (Mouse monoclonal)	Abcam	Cat# ab25630, RRID:AB_470708	Immunohistochemistry (1:100)/Western blot (1:1000)
Antibody	anti-Human CD107a (Rat monoclonal)	Abcam	Cat# ab25245, RRID:AB_449893	Immunofluorescent staining (1:100)
Antibody	anti-Human CD146-FITC (Mouse monoclonal)	Bio-Rad	Cat# MCA2141F, RRID:AB_324069	Flow cytometry (1:100)
Antibody	anti-Human CD146 (Rabbit monoclonal)	Abcam	Cat# ab75769, RRID:AB_2143375	Immunofluorescent staining (1:100)
Antibody	anti-human GAPDH (Rabbit monoclonal)	Cell Signaling Technology	Cat# 5174, RRID:AB_10622025	Western blot (1:1000)
Antibody	anti-human Gli1 (Rabbit polyoclonal)	Abcam	Cat# ab49314, RRID:AB_880198	Immunofluorescent staining (1:100)
Antibody	anti-human Nuclei (Mouse monoclonal)	Sigma-Aldrich	Cat# MAB1281, RRID:AB_94090	Immunofluorescent staining (1:500)
Antibody	anti-human Osteocalcin (Rabbit polyclonal)	Abcam	Cat# ab93876, RRID:AB_10675660	Immunofluorescent staining (1:100)
Antibody	anti-human αSMA (Rabbit polyclonal)	Abcam	Cat# ab21027, RRID:AB_1951138	Immunofluorescent staining (1:100)
Antibody	Anti-rabbit IgG, HRP-linked (Goat polyclonal)	Cell Signaling Technology	Cat# 7074, RRID:AB_2099233	Western blot (1:5000)
Antibody	Anti-mouse IgG, HRP-linked (Horse polyclonal)	Cell Signaling Technology	Cat# 7076, RRID:AB_330924	Western blot (1:5000)
Antibody	anti-mouse IgG H and L-AF488 (Goat polyclonal)	Abcam	Cat# ab150117, RRID:AB_2688012	Immunofluorescent staining (1:200)
Antibody	anti-rabbit IgG H and L-AF488 (Goat polyclonal)	Abcam	Cat# ab150077, RRID:AB_2630356	Immunofluorescent staining (1:200)
Antibody	anti-rabbit IgG H+L-DyLight 594 (Goat)	Vector Laboratories	Cat# DI-1594, RRID:AB_2336413	Immunofluorescent staining (1:200)
Antibody	anti-goat IgG H and L-AF647 (Donkey polyclonal)	Abcam	Cat# ab150135, RRID:AB_2687955	Immunofluorescent staining (1:200)
Antibody	anti-mouse IgG H and L-AF647 (Goat polyclonal)	Abcam	Cat# ab150119, RRID:AB_2811129	Immunofluorescent staining (1:200)
Antibody	anti-rabbit IgG H and L-AF647 (Goat polyclonal)	Abcam	Cat# ab150079, RRID:AB_2722623	Immunofluorescent staining (1:200)
Antibody	anti-rat IgG H and L-AF647 (Goat polyclonal)	Abcam	Cat# ab150167, RRID:AB_2864291	Immunofluorescent staining (1:200)
Antibody	ImmPRESS-AP Anti-Mouse Reagent antibody (Horse)	Vector Laboratories	Cat# MP-5402, RRID:AB_2336535	Immunohistochemistry (200 μl)
Sequence-based reagent	*ACAN*_F	This paper	PCR primers	AGGCTGGGGAGAGAACTGAAAAG
Sequenced-based reagent	*ACAN*_R	This paper	PCR primers	GCTCACAATGGGGTATCTGACAG
Sequenced-based reagent	*ACTB*_F	This paper	PCR primers	CTGGAACGGTGAAGGTGACA
Sequenced-based reagent	*ACTB*_R	This paper	PCR primers	AAGGGACTTCCTGTAACAATGCA
Sequenced-based reagent	*ALPL*_F	This paper	PCR primers	ACCACCACGAGAGTGAACCA
Sequenced-based reagent	*ALPL*_R	This paper	PCR primers	CGTTGTCTGAGTACCAGTCCC
Sequenced-based reagent	*COL2A1*_F	This paper	PCR primers	CCGCGGTGAGCCATGATTCG
Sequenced-based reagent	*COL2A1*_R	This paper	PCR primers	CAGGCCCAGGAGGTCCTTTGGG
Sequenced-based reagent	*COMP*_F	PMID:23382851	PCR primers	CAACTGTCCCCAGAAGAGCAA
Sequenced-based reagent	*COMP*_R	PMID:23382851	PCR primers	TGGTAGCCAAAGATGAAGCCC
Sequenced-based reagent	*FABP4*_F	This paper	PCR primers	ACGAGAGGATGATAAACTGGTGG
Sequenced-based reagent	*FABP4*_R	This paper	PCR primers	GCGAACTTCAGTCCAGGTCAAC
Sequenced-based reagent	*GAPDH*_F	PMID:31482845	PCR primers	CTGGGCTACACTGAGCACC
Sequenced-based reagent	*GAPDH_R*	PMID:31482845	PCR primers	AAGTGGTCGTTGAGGGCAATG
Sequenced-based reagent	*LAMP1_F*	This paper	PCR primers	GTCTTCTTCGTGCCGGCGT
Sequenced-based reagent	*LAMP1_R*	This paper	PCR primers	GCAGGTCAAAGGTCATGTTCTT
Sequenced-based reagent	*LPL_F*	This paper	PCR primers	TTGCAGAGAGAGGACTCGGA
Sequenced-based reagent	*LPL_R*	This paper	PCR primers	GGAGTTGCACCTGTATGCCT
Sequenced-based reagent	*SPP1_F*	This paper	PCR primers	CCTCCTAGGCATCACCTGTG
Sequenced-based reagent	*SPP1_R*	This paper	PCR primers	CCACACTATCACCTCGGCC
Sequenced-based reagent	*RUNX2_F*	PMID:31482845	PCR primers	TGGTTACTGTCATGGCGGGTA
Sequenced-based reagent	*RUNX2_R*	PMID:31482845	PCR primers	TCTCAGATCGTTGAACCTTGCTA
Sequenced-based reagent	*PPARG_F*	This paper	PCR primers	GACAGGAAAGACAACAGACAAATC
Sequenced-based reagent	*PPARG_R*	This paper	PCR primers	GGGGTGATGTGTTTGAACTTG
Sequenced-based reagent	*RUNX2_F*	This paper	PCR primers	TGGTTACTGTCATGGCGGGTA
Sequenced-based reagent	*RUNX2_R*	This paper	PCR primers	TCTCAGATCGTTGAACCTTGCTA
Sequenced-based reagent	*SOX9_F*	This paper	PCR primers	GAGGAAGTCGGTGAAGAACG
Sequenced-based reagent	*SOX9_R*	This paper	PCR primers	ATCGAAGGTCTCGATGTTGG
Commercial assay or kit	Leukocyte Alkaline Phosphatase Kit	Sigma-Aldrich	Cat#85L2-1KT	
Chemical compound, drug	Vacuolin-1	Sigma-Aldrich	Cat#673000	
Chemical compound, drug	TransIT-LT1 Transfection Reagent	Mirus Bio	Cat#MIR2300	Transfection
Software, algorithm	FlowJo	FlowJo	RRID:SCR_008520	
Software, algorithm	ImageJ	NIH	RRID:SCR_003070	
Software, algorithm	Prism	GraphPad	RRID:SCR_002798	
Software, algorithm	Seurat	Seurat	RRID:SCR_016341	
Software, algorithm	Monocle 3	Monocle	RRID:SCR_018685	
Software, algorithm	Partek Genomics Suite	Partek	RRID:SCR_011860	
Software, algorithm	Spotfire	Spotfire	RRID:SCR_008858	
Software, algorithm	Ingenuity Pathway Analysis	QIAGEN	RRID:SCR_008653	
Software, algorithm	SPSS	SPSS	RRID:SCR_002865	
Other	DAPI stain	Vector Laboratories	Cat# H-1500, RRID:AB_2336788	
Other	Vector Red Substrate Kit	Vector Laboratories	Cat# SK-5100, RRID:AB_2336847	
