# Supplementary material for: Lysosomal protein surface expression discriminates fat- from bone-forming human mesenchymal precursor cells
Source: eLife. 2020 Oct 12;9:e58990. doi: 10.7554/eLife.58990 (PMC7550188; doi:10.7554/eLife.58990)
Supplement: Supplementary file 1. — Percentages are based on cell frequency within the PI- cell population. [file elife-58990-supp1.docx]

**Supplementary File 1.** Frequency of CD107a^low^CD31^-^CD45^-^ or CD107a^high^CD31^-^CD45^-^ cells by FACS sorting. Percentages are based on cell frequency within the PI- cell population.

| **Sample no.** | **Donor area** | **Gender** | **Age** | **BMI** | **CD31^-^CD45^-^CD107a^low^ population** | **CD31^-^CD45^-^CD107a^high^ population** |
| --- | --- | --- | --- | --- | --- | --- |
|  |  |  |  |  | % of PI^-^ cells | % of PI^-^ cells |
| 1 | abdomen | F | 54 | 30 | 40.53 | 3.78 |
| 2 | abdomen | F | 55 | 32 | 38.16 | 7.92 |
| 3 | abdomen | F | 39 | 25 | 35.00 | 4.30 |
| 4 | thigh | F | 42 | 28 | 38.00 | 9.07 |
| 5 | abdomen/flank | F | 45 | 33 | 16.78 | 5.98 |
| 6 | abdomen | F | 38 | 38 | 43.10 | 2.92 |
| 7 | abdomen | F | 52 | 25 | 38.30 | 2.85 |
| 8 | abdomen | F | 42 | 28 | 20.1 | 4.74 |
| **Ave** | | | | | 33.75 | 5.20 |
| **SD** | | | | | 9.76 | 2.29 |
