# Supplementary material for: Lysosomal protein surface expression discriminates fat- from bone-forming human mesenchymal precursor cells
Source: eLife. 2020 Oct 12;9:e58990. doi: 10.7554/eLife.58990 (PMC7550188; doi:10.7554/eLife.58990)
Supplement: Supplementary file 2. [file elife-58990-supp2.docx]

**Supplementary File 2.** CD34 frequency among freshly isolated CD107a^low^ and CD107a^high^ cells.

| CD34 frequency among CD31^-^CD45^-^CD107a^low/high^ | | |
| --- | --- | --- |
| Cell batch | **CD107a^low^** | **CD107a^high^** |
| 1 | 59.1 | 44 |
| 2 | 65.4 | 79.5 |
| 3 | 87.5 | 39.4 |
| 4 | 71.5 | 46.9 |
| Ave | 70.88 | 52.45 |
| SD | 12.18 | 18.30 |
| p-value | 0.1447 | |
