# Supplementary material for: Lysosomal protein surface expression discriminates fat- from bone-forming human mesenchymal precursor cells
Source: eLife. 2020 Oct 12;9:e58990. doi: 10.7554/eLife.58990 (PMC7550188; doi:10.7554/eLife.58990)
Supplement: Supplementary file 3. [file elife-58990-supp3.docx]

**Supplementary File 3.** CD146 frequency among freshly isolated CD107a^low^ and CD107a^high^ cells.

| CD146 frequency among CD31^-^CD45^-^CD107a^low/high^ | | |
| --- | --- | --- |
| Cell batch | **CD107a^low^** | **CD107a^high^** |
| 1 | 10.7 | 2.14 |
| 2 | 12.2 | 5.14 |
| 3 | 1.37 | 6.57 |
| 4 | 3.48 | 4.03 |
| Ave | 6.94 | 4.47 |
| SD | 5.32 | 1.87 |
| p-value | 0.4149 | |
