# Supplementary material for: Lysosomal protein surface expression discriminates fat- from bone-forming human mesenchymal precursor cells
Source: eLife. 2020 Oct 12;9:e58990. doi: 10.7554/eLife.58990 (PMC7550188; doi:10.7554/eLife.58990)
Supplement: Supplementary file 4. [file elife-58990-supp4.docx]

**Supplementary File 4.** Canonical mesenchymal stem cell (MSC) markers among freshly isolated CD107a^low^ and CD107a^high^ cells.

| Markers | | Batch 1 | | | Batch 2 | | | Batch 3 | | Summary | | | | |
| --- | --- | --- | --- | --- | --- | --- | --- | --- | --- | --- | --- | --- | --- | --- |
|  | **CD107a^low^** | | **CD107a^high^** | **CD107a^low^** | | **CD107a^high^** | **CD107a^low^** | | **CD107a^high^** | **CD107a^low^** | | **CD107a^high^** | | **p-value** |
|  |  |  |  |  |  |  |  |  |  | **Ave** | **SD** | **Ave** | **SD** |  |
| CD44 | 26.5 | | 27.1 | 28.4 | | 67 | 12.4 | | 52.1 | 22.43 | 8.74 | 48.73 | 20.16 | 0.1069 |
| CD73 | 29.8 | | 32.6 | 24.6 | | 57.3 | 7.3 | | 14.6 | 20.57 | 11.78 | 34.83 | 21.44 | 0.3695 |
| CD90 | 75 | | 92.3 | 40.5 | | 82.3 | 18.1 | | 43.5 | 52.55 | 23.42 | 69.2 | 24.01 | 0.2744 |
| CD105 | 0.25 | | 10.8 | 0.91 | | 6.6 | 0.15 | | 6.96 | 0.44 | 0.42 | 8.12 | 2.33 | 0.0049 |
