# Supplementary material for: Lysosomal protein surface expression discriminates fat- from bone-forming human mesenchymal precursor cells
Source: eLife. 2020 Oct 12;9:e58990. doi: 10.7554/eLife.58990 (PMC7550188; doi:10.7554/eLife.58990)
Supplement: Supplementary file 5. — Implants were placed bilaterally, with each animal receiving the same treatment on either hindlimb. [file elife-58990-supp5.docx]

**Supplementary File 5.** Animal allocation for intramuscular implantation using 12 NOD-SCID mice. Implants were placed bilaterally, with each animal receiving the same treatment on either hindlimb.

| **Cell type** | **Scaffold type and weight** | **Cell number (#)** | **Implant number (#)** |
| --- | --- | --- | --- |
| Scaffold alone (acellular control) | DBX® Putty (45 mg) | 0 | 8 |
| Human CD107a^low^CD31^-^CD45^-^ cells |  | 3.0 × 10^6^ | 8 |
| Human CD107a^high^CD31^-^CD45^-^ cells |  | 3.0 × 10^6^ | 8 |
