# Supplementary material for: Lysosomal protein surface expression discriminates fat- from bone-forming human mesenchymal precursor cells
Source: eLife. 2020 Oct 12;9:e58990. doi: 10.7554/eLife.58990 (PMC7550188; doi:10.7554/eLife.58990)
Supplement: Supplementary file 6. — Cell-augmented grafts were placed bilaterally on either side of the lumbar spine, with scaffold and cell numbers per side shown. [file elife-58990-supp6.docx]

**Supplementary File 6.** Animal allocation for posterolateral lumbar spine fusion model in athymic rats. Cell augmented grafts were placed bilaterally on either side of the lumbar spine, with scaffold and cell numbers per side shown.

| **Cell type** | **Scaffold type and weight per side** | **Cell Number (#) per side** | **Animal Number (#)** |
| --- | --- | --- | --- |
| Scaffold alone (acellular control) | DBX® Putty (230 mg) | 0 | 6 |
| Human CD107a^low^CD31^-^CD45^-^ cells |  | 7.5 × 10^5^ | 8 |
| Human CD107a^high^CD31^-^CD45^-^ cells |  | 7.5 × 10^5^ | 8 |
