# Supplementary material for: Lysosomal protein surface expression discriminates fat- from bone-forming human mesenchymal precursor cells
Source: eLife. 2020 Oct 12;9:e58990. doi: 10.7554/eLife.58990 (PMC7550188; doi:10.7554/eLife.58990)
Supplement: Supplementary file 7. [file elife-58990-supp7.docx]

**Supplementary File 7.** Antibodies used.

| **Antibody** | **Company** | **Catalog #** | **Use** |
| --- | --- | --- | --- |
| Mouse anti-Human CD31 | BD Pharmingen | 563653 | FACS / F |
| Mouse anti-Human CD31 | Abcam | ab24590 | IF |
| Rabbit anti-Human CD31 | Abcam | ab28364 | IF |
| Mouse anti-Human CD34 | BD Pharmingen | 562383 | F |
| Rabbit anti-Human CD34 | Abcam | ab81289 | IF |
| Mouse anti-Human CD44 | BD Pharmingen | 561289 | F |
| Mouse anti-Human CD45 | BD Pharmingen | 557833 | FACS / F |
| Mouse anti-Human CD73 | BD Pharmingen | 561014 | F |
| Mouse anti-Human CD90 | BD Pharmingen | 555595 | F |
| Mouse anti-Human CD105 | BD Pharmingen | 562380 | F |
| Mouse anti-Human CD107a | BD Pharmingen | 560664 | FACS / F / ICC |
| Mouse anti-Human CD107a | R&D | MAB4800 | IF |
| Mouse anti-Human CD107a | Abcam | ab25630 | IHC/WB |
| Rat anti-Human CD107a | Abcam | ab25245 | IF |
| Mouse anti-Human CD146 | Bio-Rad | MCA2141F | F |
| Rabbit anti-Human CD146 | Abcam | ab75769 | IF |
| Rabbit anti-Human GAPDH | Cell Signaling Technology | 5174S | WB |
| Rabbit anti-Human Gli1 | Abcam | ab49314 | IF |
| Mouse anti-Human Nuclei | Sigma | MAB1281 | IF |
| Rabbit anti-Human osteocalcin | Abcam | ab93876 | IF |
| Goat anti-Human αSMA | Abcam | ab21027 | IF |
| Goat anti-Mouse AF488 | Abcam | ab150117 | IF |
| Goat anti-Rabbit AF488 | Abcam | ab150077 | IF |
| Goat anti-Rabbit DyLight 594 | Vector Laboratories | DI-1594 | IF |
| Donkey anti-Goat AF647 | Abcam | ab150135 | IF |
| Goat anti-Mouse AF647 | Abcam | ab150119 | IF |
| Goat anti-Rabbit AF647 | Abcam | ab150079 | IF |
| Goat anti-Rat AF647 | Abcam | ab150167 | IF |
| Anti-rabbit IgG, HRP-linked Antibody | Cell Signaling Technology | 7074S | WB |
| Anti-mouse IgG, HRP-linked Antibody | Cell Signaling Technology | 7076S | WB |
| F: Flow cytometry; FACS: Fluorescent activated cell sorting; IF: Immunofluorescent staining; IHC: Immunohistochemistry; ICC: Immunocytochemistry; WB: Western blot. | | | |
