# Supplementary material for: Lysosomal protein surface expression discriminates fat- from bone-forming human mesenchymal precursor cells
Source: eLife. 2020 Oct 12;9:e58990. doi: 10.7554/eLife.58990 (PMC7550188; doi:10.7554/eLife.58990)
Supplement: Supplementary file 8. [file elife-58990-supp8.docx]

**Supplementary File 8.** Primers used.

| Genes (human) | Forward | Reverse |
| --- | --- | --- |
| *ACAN* | 5’-AGGCTGGGGAGAGAACTGAAAAG-3’ | 5’-GCTCACAATGGGGTATCTGACAG-3’ |
| *ACTB* | 5’-CTGGAACGGTGAAGGTGACA-3’ | 5’-AAGGGACTTCCTGTAACAATGCA-3’ |
| *ALPL* | 5’-ACCACCACGAGAGTGAACCA-3’ | 5’-CGTTGTCTGAGTACCAGTCCC-5’ |
| *COL2A1* | 5’-CCGCGGTGAGCCATGATTCG-3’ | 5’-CAGGCCCAGGAGGTCCTTTGGG-3’ |
| *COMP* | 5’-CAACTGTCCCCAGAAGAGCAA-3’ | 5’-TGGTAGCCAAAGATGAAGCCC-3’ |
| *FABP4* | 5’-ACGAGAGGATGATAAACTGGTGG-3’ | 5’-GCGAACTTCAGTCCAGGTCAAC-3’ |
| *GAPDH* | 5’-CTGGGCTACACTGAGCACC-3’ | 5’-AAGTGGTCGTTGAGGGCAATG-3’ |
| *LAMP1* | 5’-GTCTTCTTCGTGCCGGCGT-3’ | 5’-GCAGGTCAAAGGTCATGTTCTT-3’ |
| *LPL* | 5’-TTGCAGAGAGAGGACTCGGA-3’ | 5’-GGAGTTGCACCTGTATGCCT-3’ |
| *PPARG* | 5’-GACAGGAAAGACAACAGACAAATC-3’ | 5’-GGGGTGATGTGTTTGAACTTG-3’ |
| *RUNX2* | 5’-TGGTTACTGTCATGGCGGGTA-3’ | 5’-TCTCAGATCGTTGAACCTTGCTA-3’ |
| *SOX9* | 5’-GAGGAAGTCGGTGAAGAACG-3’ | 5’-ATCGAAGGTCTCGATGTTGG-3’ |
| *SPP1* | 5’‐CCTCCTAGGCATCACCTGTG‐3’ | 5’‐CCACACTATCACCTCGGCC‐3’ |
