# Supplementary material for: Lysosomal protein surface expression discriminates fat- from bone-forming human mesenchymal precursor cells
Source: eLife. 2020 Oct 12;9:e58990. doi: 10.7554/eLife.58990 (PMC7550188; doi:10.7554/eLife.58990)

**Supplementary File 9.** Uncropped versions of representative western blot images from Figure 3–figure supplement 4B.


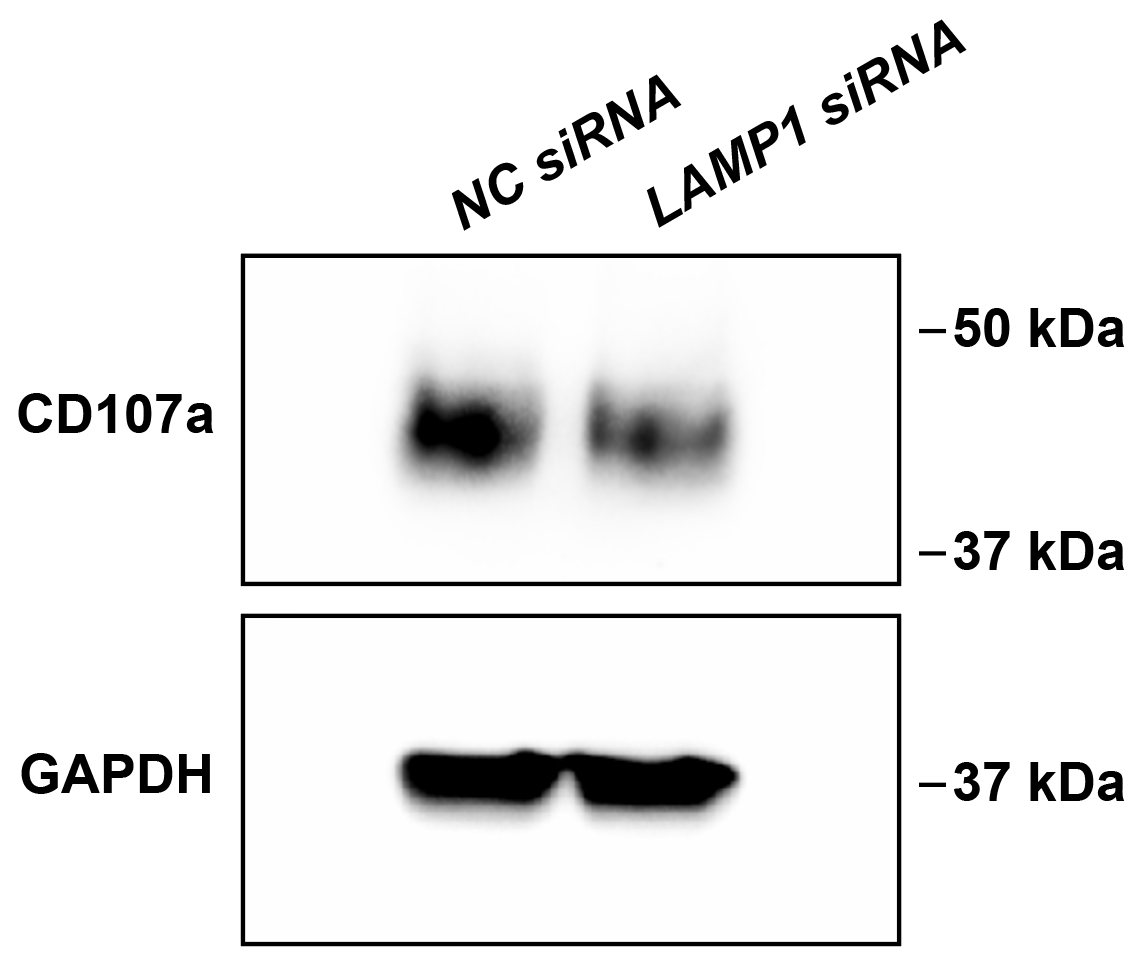

Supplement: Supplementary file 9. [file elife-58990-supp9.docx]
